# P2X7 receptor drives Th1 cell differentiation and controls the follicular helper T cell population to protect against *Plasmodium chabaudi* malaria

**DOI:** 10.1371/journal.ppat.1006595

**Published:** 2017-08-31

**Authors:** Érika Machado de Salles, Maria Nogueira de Menezes, Renan Siqueira, Henrique Borges da Silva, Eduardo Pinheiro Amaral, Sheyla Inés Castillo-Méndez, Isabela Cunha, Alexandra dos Anjos Cassado, Flávia Sarmento Vieira, David Nicholas Olivieri, Carlos Eduardo Tadokoro, José Maria Alvarez, Robson Coutinho-Silva, Maria Regina D’Império-Lima

**Affiliations:** 1 Departamento de Imunologia, Instituto de Ciências Biomédicas, Universidade de São Paulo, São Paulo, Brazil; 2 Department of Laboratory Medicine and Pathology, Center of Immunology, University of Minnesota, Minneapolis, Minnesota, United States; 3 Escuela Superior de Enxeñaría Informática, Universidad de Vigo, Vigo, Spain; 4 Universidade Vila Velha, Vila Velha, ES, Brazil; 5 Programa de Imunobiologia, Instituto de Biofísica Carlos Chagas Filho, Universidade Federal do Rio de Janeiro, Rio de Janeiro, Brazil; 6 Instituto Nacional de Ciência e Tecnologia para Pesquisa Translacional em Saúde e Meio Ambiente da Região Amazônica, Rio de Janeiro, Brazil; Francis Crick Institute, UNITED KINGDOM

## Abstract

A complete understanding of the mechanisms underlying the acquisition of protective immunity is crucial to improve vaccine strategies to eradicate malaria. However, it is still unclear whether recognition of damage signals influences the immune response to *Plasmodium* infection. Adenosine triphosphate (ATP) accumulates in infected erythrocytes and is released into the extracellular milieu through ion channels in the erythrocyte membrane or upon erythrocyte rupture. The P2X7 receptor senses extracellular ATP and induces CD4 T cell activation and death. Here we show that P2X7 receptor promotes T helper 1 (Th1) cell differentiation to the detriment of follicular T helper (Tfh) cells during blood-stage *Plasmodium chabaudi* malaria. The P2X7 receptor was activated in CD4 T cells following the rupture of infected erythrocytes and these cells became highly responsive to ATP during acute infection. Moreover, mice lacking the P2X7 receptor had increased susceptibility to infection, which correlated with impaired Th1 cell differentiation. Accordingly, IL-2 and IFNγ secretion, as well as T-bet expression, critically depended on P2X7 signaling in CD4 T cells. Additionally, P2X7 receptor controlled the splenic Tfh cell population in infected mice by promoting apoptotic-like cell death. Finally, the P2X7 receptor was required to generate a balanced Th1/Tfh cell population with an improved ability to transfer parasite protection to CD4-deficient mice. This study provides a new insight into malaria immunology by showing the importance of P2X7 receptor in controlling the fine-tuning between Th1 and Tfh cell differentiation during *P*. *chabaudi* infection and thus in disease outcome.

## Author summary

Malaria still causes the death of approximately half a million people yearly despite efforts to develop vaccines. The ability of *Plasmodium* parasites to survive the immune effector mechanisms indicates how suitable the immune response must be to eliminate the infection. CD4 T cells have a dual role in protection against blood-stage malaria by producing IFNγ and helping B cells to secrete antibodies. Infected erythrocytes release adenosine triphosphate (ATP), a damage signal that can be recognized by purinergic receptors. Among them, the P2X7 receptor senses extracellular ATP and induces CD4 T cell activation and death. Here, we evaluated the role of P2X7 receptor in the CD4 T cell response during blood-stage *Plasmodium chabaudi* malaria. We observed that the selective expression of P2X7 receptor in CD4 T cells was required for T helper 1 (Th1) cell differentiation, contributing to IFNγ production and parasite control. In contrast, we found an increase in follicular T helper (Tfh) cell population, germinal center reaction and anti-parasite antibody production in the absence of the P2X7 receptor. Our findings provide mechanistic insights into malaria pathogenesis by demonstrating the importance of damage signals for the fine-tuning between Th1 and Tfh cell populations and thus for the outcome of the disease.

## Introduction

Despite efforts to develop vaccines and antimalarial drugs, *Plasmodium* infection still causes the death of about half a million people yearly [1]. The most prevalent *Plasmodium* species, *Plasmodium falciparum* and *Plasmodium vivax*, persist for very long time periods in the bloodstream of infected individuals. Parasite persistence is also ensured by repeated re-infections in hyperendemic areas. Although the major clinical manifestations of the disease attenuate after a few malaria episodes, repeated exposure to the parasite over several years is required to control parasite population growth [2]. Furthermore, protective immunity is usually lost in the absence of continued exposure to the parasite [3]. The ability to survive the effector mechanisms of innate immunity and to evade the acquired immune response for long periods shows how difficult it is to combat malaria and how appropriate the immune response must be to eliminate the parasite. Therefore, a complete understanding of the mechanisms underlying the acquisition of protective immunity is crucial to improve vaccine strategies to eradicate malaria. Particularly concerning the stimulatory signaling required for optimal activation of CD4 T cells, which have a central role in protection against malaria by producing IFNγ and helping B cells to secrete antibodies [4], [5].

The intraerythrocytic cycle of *P*. *falciparum* and *P*. *vivax* has a synchronic periodicity and, consequently, the delivery of immune stimulatory molecules and subsequent fever episodes occur periodically after the rupture of infected red blood cells (iRBCs). It has been shown that parasite components, such as glycosylphosphatidylinositol (GPIs)-anchored molecules and DNA from *P*. *falciparum*, activate macrophages through the toll-like receptor (TLR)1/TLR2 and TLR9 signaling, respectively [6]. Nevertheless, it is still unclear whether recognition of damage signals contributes to activating the immune system in individuals suffering from malaria. During pathogenic infection and tissue injury, nucleic acids and their metabolites are released from dead cells and induce inflammatory and reparatory responses [7], [8]. Adenosine triphosphate (ATP) is released passively from necrotic cells and through pannexin-1 hemichannels from apoptotic cells [9]. ATP accumulates in iRBCs and is released into the extracellular milieu through ion channels in the erythrocyte membrane or upon iRBC rupture [10], [11]. ATP is also released through pannexin-1 hemichannels in the immune synapsis formed between T cells and antigen presenting cells (APCs), triggering ATP-gated ionotropic P2X receptors that promote IL-2 secretion and T cell proliferation [12]. Unlike P2X1 and P2X4 receptors, which translocate into the immune synapsis, the P2X7 receptor remains uniformly distributed across the cell surface, allowing T cells to sense environmental ATP. The P2X7 receptor is activated only at high ATP concentrations [13], hence it may be particularly important to help T cells distinguish tissue-damaging infections from quiescent infections or reminiscent antigens from a previous infection. Transient P2X7 activation promotes T cell response due to the formation of a non-selective cation channel that allows calcium influx [14]. However, sustained signaling induces the formation of large transmembrane pores and, consequently, leads to loss of membrane integrity and T cell death [15].

Although P2X7 signaling has important consequences for T cell biology, few studies have addressed the direct effects of this signaling pathway on T cell fate *in vivo*. Interestingly, the *P2rx7* gene is highly expressed in follicular helper T cells (Tfh) located in Peyer's patches and the P2X7 receptor critically controls their numbers and, consequently, the production of IgA against gut commensals [16]. Increased *P2rx7* expression is also a feature of regulatory T cells (Treg cells); ATP stimulation inhibits Treg cell generation and suppressive activity through the P2X7 receptor [17]. Moreover, it has been shown that P2X7 activation by extracellular (eATP) can be abrogated by CD39 (nucleoside triphosphate diphosphohydrolase-1), an ectoATPase that degrades ATP or adenosine diphosphate (ADP) to adenosine monophosphate (AMP). CD39 is constitutively expressed on Treg cell surface [18], providing protection against ATP-induced cell death [19]. CD39 and CD73 (ecto-5’-nucleotidase) also contribute to the suppressive activity of Treg cells [18]. CD73 hydrolyses extracellular AMP to adenosine, which is an important physiological regulator of the immune response [20].

In this study, we investigated using the blood-stage *Plasmodium chabaudi* (*Pc*) murine infection model whether P2X7 signaling contributes to CD4 T cell subset differentiation in malaria. The infection with synchronic *Pc* parasites develops from an acute phase to a long-lasting chronic phase, which accurately reproduces several aspects of human malaria [21]. IFNγ production is associated with the development of protective immunity [22], [23]. A major source of IFNγ during acute *Pc* infection is class II MHC (major histocompatibility complex)-restricted CD4 T cells, which also help B cells to secrete antibodies [24]. The complete elimination of chronic parasitemia and protection against reinfection require Th1 cells [25], [26], which are particularly important in ensuring long-term strain-transcending immunity [27]. A Th1 cell population co-expressing IFNγ and IL-10 also plays a key role in protecting against severe malaria pathology [28]. Furthermore, Tfh cells provide critical help to B cells to produce high affinity antibodies [29], [30], and have been the focus of recent studies in murine and human malaria [31]. Tfh cells are implicated in protection against both *Pc* and *Plasmodium yoelii* 17XNL parasites [32]-[34].

Our results suggest that P2X7 receptor is required for Th1 cell differentiation during *Pc* infection but it also controls the Tfh cell population. Using adoptive transfer experiments, we showed that the selective absence of the P2X7 receptor in CD4 T cells is sufficient to impair Th1 cell differentiation and increase the Tfh cell population. Evidencing the importance of the fine-tuning between Th1 and Tfh cell populations in the control of *Pc* infection, the balanced Th1/Tfh cell population that differentiated in the presence of P2X7 receptor displayed higher ability to transfer protection to CD4-deficient mice than the increased Tfh cell population developed in its absence. The present study adds novel information on the malaria immunology field by demonstrating the critical role of the P2X7 receptor for the outcome of *Pc* infection by promoting Th1 cell differentiation to the detriment of Tfh cells.

## Results

### P2X7 receptor plays opposing roles in the protection against *Pc* and *P*. *yoelii* 17XNL malaria

**Fig 1:**
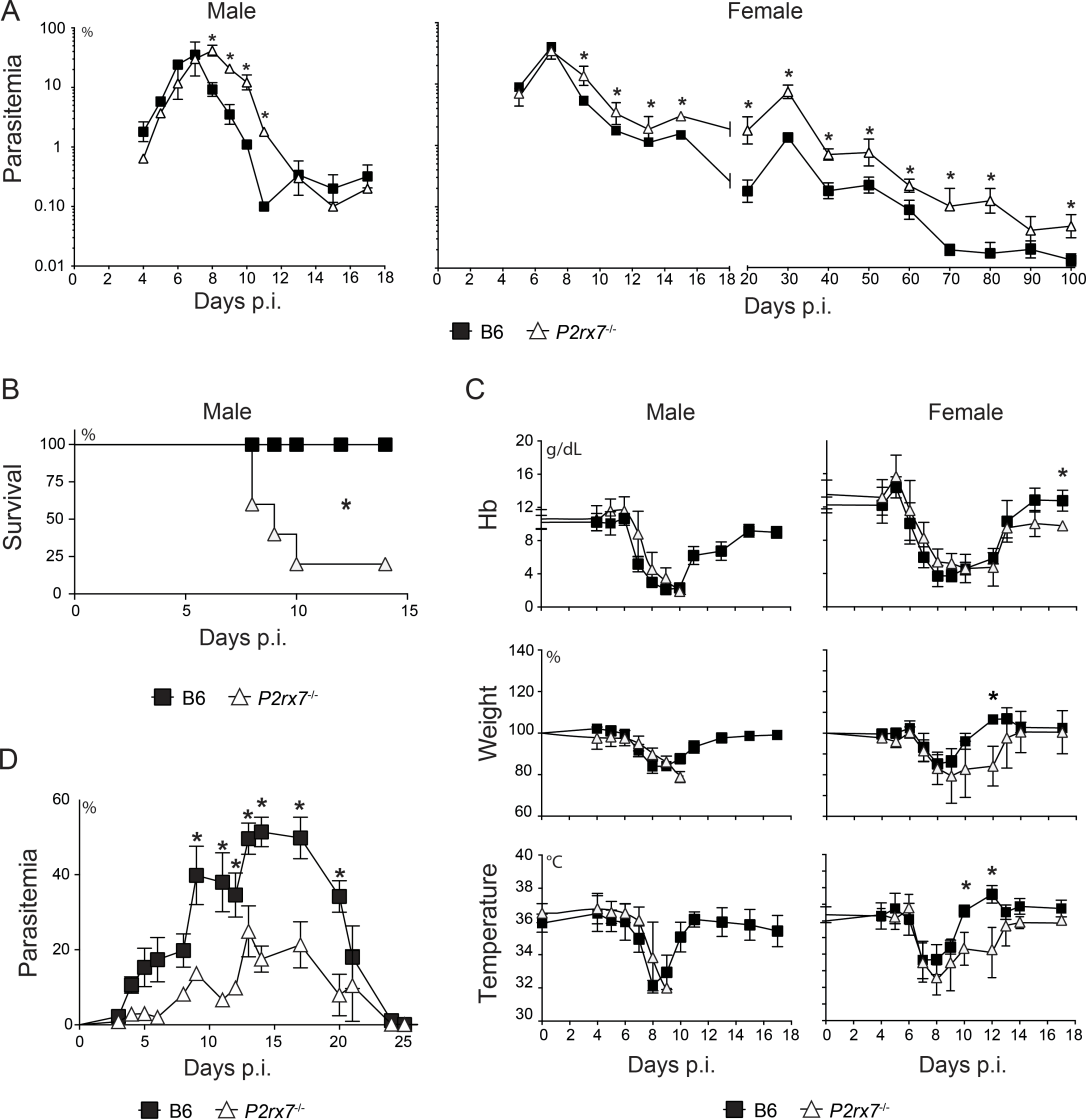
Parasitemia and clinical parameters in B6 and *P2rx7*^-/-^ mice infected with *Pc* and P. *yoelii* 17NL parasites. (A-C) B6 and *P2rx7*^-/-^ mice were infected with 1 × 10^6^ *Pc*-iRBCs. The data were expressed as means ± SD (*n* = 4–5) of one representative experiment out of three. Significant differences were observed for the (*) B6 and *P2rx7*^-/-^ groups with *p* < 0.05, using (A and C) the Mann Whitney U test or (B) the long-rank test. (A) Parasitemia curves are shown. (B) Survival curves are shown. (C) Hemoglobin serum (Hb) concentration, body weight and body temperature were monitored daily. Variation in body weight relative to day 0 is shown. (D) B6 and *P2rx7*^-/-^ mice were infected with 1 × 10^5^ *P*. *yoelii* 17XNL-iRBCs. The data were expressed as means ± SD (*n* = 4–5) of one representative experiment out of three. Significant differences were observed for the (*) B6 and *P2rx7*^-/-^ groups with *p* < 0.05, using the Mann Whitney U test. Parasitemia curves are shown.

To investigate the participation of the P2X7 receptor in blood-stage *Pc* malaria, disease progression was evaluated in C57BL/6 (B6) and *P2rx7*^-/-^ mice. The absence of the P2X7 receptor led to a worsening of the disease in infected females and males. Infected *P2rx7*^-/-^ males showed higher parasitemias than their B6 counterparts, and 80% of the animals died during acute infection (Fig 1A and 1B). The disease developed similarly in both female groups up to day 7 p.i.; however, after this period, *P2rx7*^-/-^ mice had impaired parasitemia control and limited recovery of clinical parameters (i.e. anemia, weight loss and hypothermia) (Fig 1C). *P2rx7*^-/-^ females also exhibited higher chronic parasitemias than B6 females (Fig 1A).

To determine whether the protection induced by the P2X7 receptor is a general phenomenon in murine malaria, we assessed the course of *P*. *yoelii* 17XNL infection in B6 and *P2rx7*^-/-^ mice. It has been shown previously that, in contrast to *Pc* infection, the lack of IFNγ modestly affects the control of *P*. *yoelii* 17XNL infection [35], whereas antibody deficiency leads to a lethal outcome [32]. Unlike the case of *Pc* malaria, more control of *P*. *yoelii* 17XNL parasites was observed in *P2rx7*^-/-^ mice than in B6 mice, apparent from day 9 to 20 p.i. when mice of both groups controlled the parasitemia (Fig 1D).

Taken together, we showed that *Pc* infection control relies on P2X7 signaling; instead, the presence of P2X7 signaling results in increased peak parasitemia during *P*. *yoelii* 17XNL infection. These results suggest that P2X7-mediated eATP sensing might play a role in controlling the balance between IFNγ- and antibody-mediated immune responses to malaria.

### P2X7 receptor is required for CD4 T cell activation and antibody production during acute *Pc* malaria

**Fig 2:**
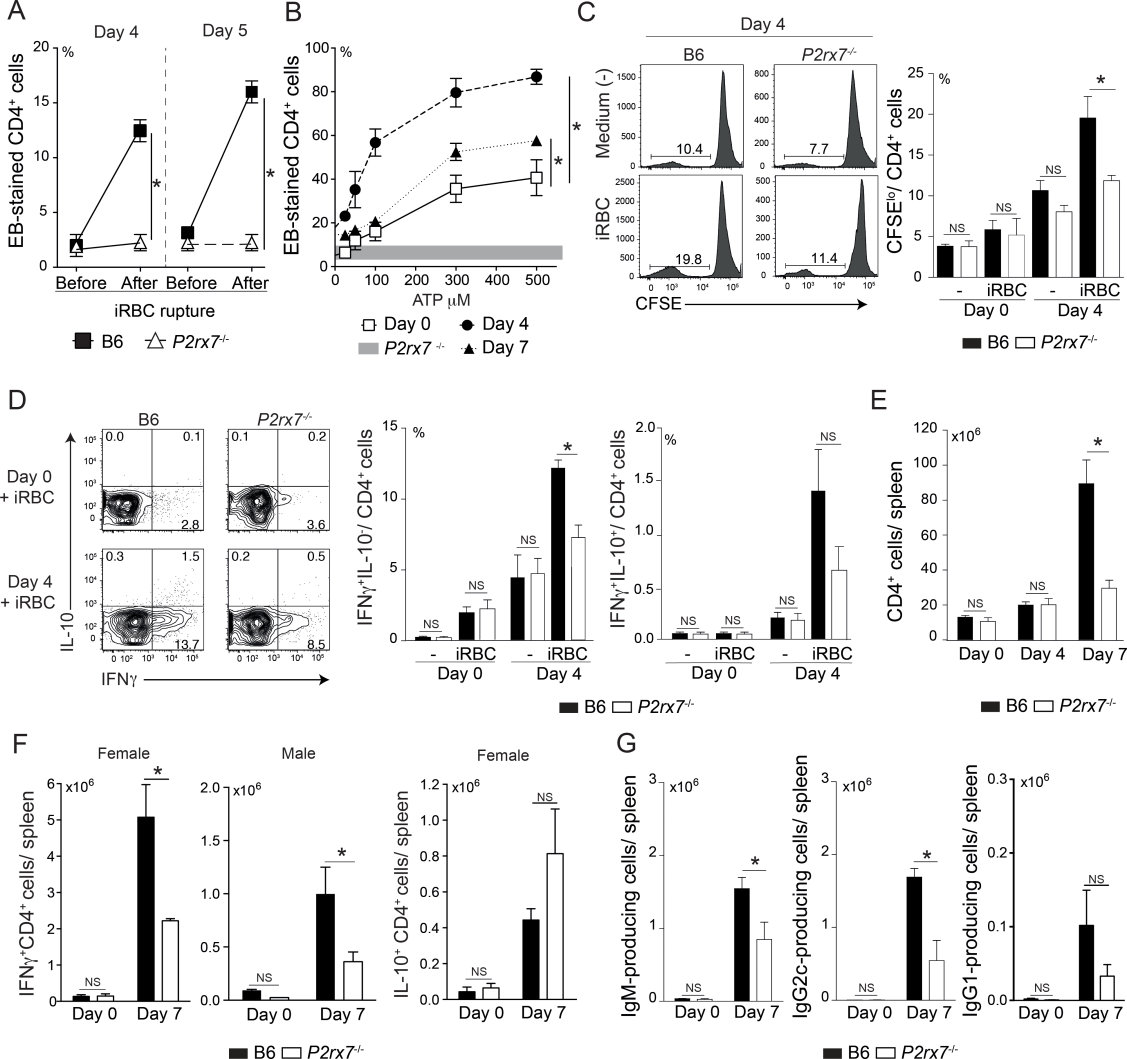
Splenic CD4 T cell responses in acutely infected B6 and *P2rx7*^-/-^ mice. (A-G) B6 and *P2rx7*^-/-^ mice were analyzed at 4, 5 and 7 days p.i. with 1 × 10^6^ *Pc*-iRBCs. Naïve mice were used as controls (day 0). Female mice were used in the experiments with the exception that females and males were compared. The data were expressed as means ± SD (*n* = 3–4) of one representative experiment out of three. Significant differences were observed for the (*) indicated groups with *p* < 0.05, using the Mann Whitney U test (NS, not significant). (A) EB-stained CD4^+^ blood cell percentages were determined by flow cytometry, before and after iRBC rupture. The blood samples were collected at 9 a.m. (1.3 ± 0.3% iRBCs at 4 days p.i. and 7.6 ± 0.3% iRBCs at 5 days p.i.; >95% trophozoites and schizonts) and 2 p.m. (7.3 ± 1.2% iRBCs at 4 days p.i. and 15.0 ± 3.7% iRBCs, at 5 days p.i.; >95% ring forms). (B) Splenocytes were stimulated or not with 25–500 μM ATP. EB-stained CD4^+^ cell percentages were determined by flow cytometry. Horizontal gray bar represents the confidence interval obtained with *P2rx7*^-/-^ cells. (C) CFSE-stained CD4^+^ cells and APCs (splenocytes from naïve nude mice), at a 1:1 ratio, were stimulated or not with iRBCs (1 splenocyte/ 4 iRBCs). Histograms show CFSE-stained CD4^+^ cells. CFSE^lo^CD4^+^ cell percentages are shown in the column bar graph. (D) Splenocytes were stimulated or not with iRBCs (1 splenocyte/ 3 iRBCs). Contour plots show intracellular IFNγ- and IL-10-stained CD4^+^ cells. IFNγ^+^IL-10^-^ and IFNγ^+^IL-10^+^ cell percentages in CD4^+^ cells are shown in the column bar graphs. (E) CD4^+^ cell numbers per spleen were determined by flow cytometry. (F) IFNγ^+^CD4^+^ and IL-10^+^CD4^+^ cell numbers per spleen were determined by flow cytometry. (G) IgM-, IgG2c- and IgG1-secreting cell numbers per spleen were determined by ELISPOT assay.

To determine whether the amount of ATP released during iRBC lysis is sufficient for P2X7 activation, P2X7-associated pore formation was evaluated by ethidium bromide (EB) uptake in blood CD4 T cells, which were obtained before and after iRBC lysis during a synchronized parasite cycle (S1A Fig). This assay explores the P2X7-mediated formation of large transmembrane pores after sustained ATP stimulation, which allows EB to enter the cell and stain the nucleus [13]. The EB staining in B6 CD4 T cells was increased after the rupture of iRBCs at 4 and 5 days p.i., and this effect was abolished in *P2rx7*^-/-^ CD4 T cells (Fig 2A). Accordingly, higher ATP serum levels were detected after iRBC lysis (S1B Fig). The effects of acute *Pc* infection on the splenic CD4 T cell response to eATP were then assessed *in vitro* at 4 and 7 days p.i., representing the interval between CD4 T cell proliferation and IFNγ secretion [24]. A marked increase in ATP-induced P2X7-mediated pore formation was observed in CD4 T cells at 4 days p.i. compared with that in non-infected controls (Fig 2B). Although the B6 CD4 T cell response to ATP was drastically reduced at day 7 p.i. compared with that at day 4 p.i., it remained augmented in relation to that of non-infected controls.

The splenic CD4 T cell response to *Pc* infection was then compared in B6 and *P2rx7*^-/-^ mice. CD4 T cells at day 4 p.i. were stimulated *in vitro* with iRBCs, mimicking the *in vivo* condition in which parasite antigens are available along with ATP released by the iRBCs; the responses were evaluated after 72 h of culture, a time point that corresponds to peak proliferation and IFNγ production [24]. *P2rx7*^-/-^ CD4 T cells proliferated less than B6 CD4 T cells in the presence of iRBCs and splenocytes from naïve nude mice, as a source of APCs expressing the P2X7 receptor (Fig 2C). IFNγ production was also reduced in iRBC-stimulated *P2rx7*^-/-^ CD4 T cells, whereas IL-10 was produced at low levels regardless of P2X7 expression (Fig 2D). The treatment with apyrase (ATP diphosphohydrolase) or brilliant blue G (BBG, P2X7 antagonist) inhibited CD4 T cell proliferation and IFNγ production, confirming the involvement of ATP and P2X7 receptor (S2A and S2B Fig). Accordingly, the lack of the P2X7 receptor drastically impaired the *in vivo* expansion of the splenic CD4 T cell population during acute *Pc* infection (Fig 2E). Moreover, at 7 days p.i., the IFNγ^+^CD4^+^ cell number per spleen was higher in B6 mice than in *P2rx7*^-/-^ mice (Fig 2F). Explaining the gender influence in the susceptibility to acute *Pc* infection, B6 and *P2rx7*^-/-^ males had lower IFNγ^+^CD4^+^ cell numbers per spleen at day 7 p.i. than female counterparts; the lowest IFNγ levels were observed for *P2rx7*^-/-^ males. The IL-10^+^CD4^+^ cell numbers were comparable in infected B6 and *P2rx7*^-/-^ mice. The immunoglobulin secretion was also reduced in acutely infected *P2rx7*^-/-^ mice compared with that in their B6 counterparts, characterized by a predominance of IgM- and IgG2c-secreting cells (Fig 2G) as previously reported [36].

Together, this data shows that P2X7 signaling at the time of *Pc*-iRBC rupture is sufficient to induce pore formation in blood CD4 T cells. They also reveal the importance of the P2X7 receptor for optimal CD4 T cell function during acute *Pc* infection.

### P2X7 receptor influences the early stages of Th1/Tfh cell differentiation during *Pc* malaria

**Fig 3:**
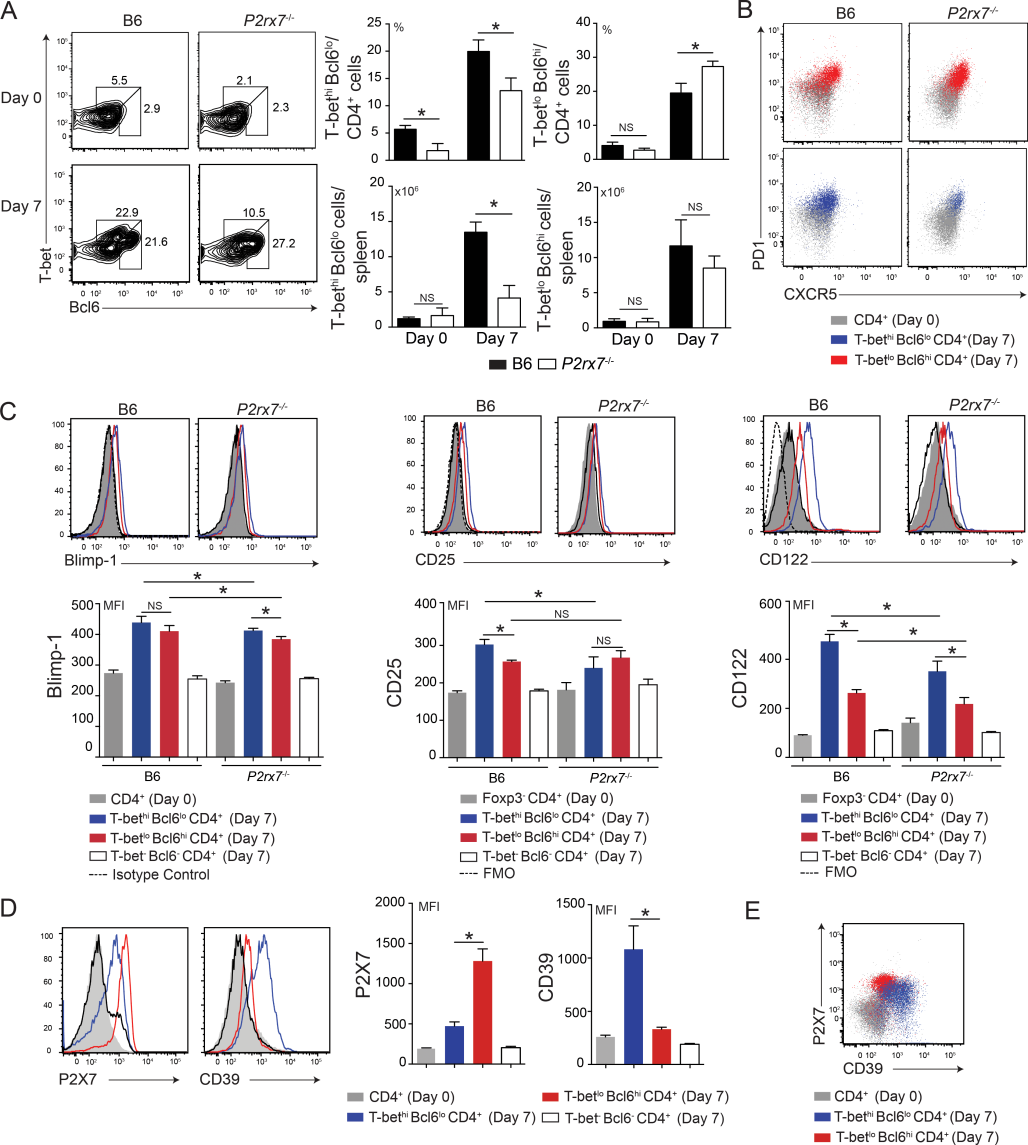
Phenotypic characterization of splenic CD4 T cells expressing T-Bet and Bcl6 in acutely infected B6 and *P2rx7*^-/-^ mice. (A-E) B6 and *P2rx7*^-/-^ female mice were analyzed at 7 days p.i. with 1 × 10^6^ *Pc*-iRBCs. Naïve mice were used as controls (day 0). The data were expressed as means ± SD (*n* = 5) of one representative experiment out of three. Significant differences were observed for the (*) indicated groups with *p* < 0.05, using (A and D) the Mann Whitney U test and (C) Kruskal-Wallis test (NS, not significant). (A) Contour plots show T-bet and Bcl6 expression in CD4^+^ cells. The gates were drawn using isotype controls (S3B Fig). T-bet^hi^Bcl6^lo^ and T-bet^lo^Bcl6^hi^ cell percentages in CD4^+^ cells and CD4^+^ cell numbers per spleen are shown in the column bar graphs. (B) Dot plots show PD1 and CXCR5 expression in CD4^+^ cells. (C) Histograms show Blimp-1, CD25 and CD122 expression in CD4^+^ or Foxp3^-^CD4^+^ (to exclude regulatory T cells), T-bet^hi^Bcl6^lo^CD4^+^, T-bet^lo^Bcl6^hi^CD4^+^ and T-bet^-^Bcl6^-^CD4^+^ cells. Fluorescence minus one (FMO) and isotype controls are shown in the histograms. The means of fluorescence intensity (MFIs) of Blimp-1, CD25 and CD122 expression are shown in the column bar graphs. (D) Histograms show P2X7 and CD39 expression in CD4^+^, T-bet^hi^Bcl6^lo^CD4^+^, T-bet^lo^Bcl6^hi^CD4^+^ and T-bet^-^Bcl6^-^CD4^+^ cells. The MFIs of P2X7 and CD39 expression are shown in the column bar graphs. (E) Dot plot shows P2X7 and CD39 expression in CD4^+^, T-bet^hi^Bcl6^lo^CD4^+^ and T-bet^lo^Bcl6^hi^CD4^+^ cells.

The low CD4 T cell proliferation in acutely infected *P2rx7*^-/-^ mice could explain their reduced IFNγ and antibody responses. However, P2X7 deficiency could also impair Th1 cell differentiation in addition to preventing CD4 T cell expansion. To evaluate this possibility, we next examined whether the P2X7 receptor influences Th1/Tfh cell differentiation during early *Pc* infection. Splenic CD4 T cells were analyzed according to the expression of the transcription factors T-bet and B cell lymphoma 6 (Bcl6), which are reciprocal regulators of Th1 and Tfh cell lineage commitment [37], [38]. At day 4 p.i., a small percentage increase in CD4 T cells expressing both T-bet and Bcl6 was observed in B6 and *P2rx7*^-/-^ mice (S3A Fig). By contrast, around 40% of the B6 CD4 T cells at 7 days p.i. showed high amounts of these transcription factors, evidenced by two distinct cell subsets (Fig 3A and S3B Fig). The proportion of T-bet^hi^Bcl6^lo^ cells was reduced in the absence of the P2X7 receptor, while that of T-bet^lo^Bcl6^hi^ cells was augmented. However, considering the low numbers of CD4 T cells per spleen in *P2rx7*^-/-^ mice at day 7 p.i. (Fig 2E), P2X7 deficiency led to a decrease in the T-bet^hi^Bcl6^lo^ cell population, but did not affect the T-bet^lo^Bcl6^hi^ cell population. Both CD4 T cell subsets showed higher expression of programmed cell death-1 (PD1) and C-X-C chemokine receptor type 5 (CXCR5) than CD4 T cells from non-infected mice (Fig 3B). However, PD1 expression in these cells at day 7 p.i. was lower than that in fully differentiated Tfh cells at 20 days p.i. (S3C Fig). Both T-bet^hi^Bcl6^lo^ and T-bet^lo^Bcl6^hi^ cells from infected B6 mice also expressed high levels of the transcription factor B lymphocyte-induced maturation protein-1 (Blimp-1) (Fig 3C, left panels), which is a strong negative regulator of Tfh cell differentiation [37]. Lower Blimp-1 expression was observed for both CD4 T cell subsets in the absence of P2X7 receptor. We also assessed the expression of IL-2 receptor α (CD25) and β (CD122) chains; effector memory Th1 cells are produced from early CD4 T cell population expressing this receptor in *L*. *monocytogenes* infection [39]. Both CD25 and CD122 were preferentially increased in T-bet^hi^Bcl6^lo^ cells than in T-bet^lo^Bcl6^hi^ cells from infected B6 mice; these molecules were expressed at lower levels in T-bet^hi^Bcl6^lo^ cells from infected *P2rx7*^-/-^ mice (Fig 3C, middle and right panels). At 7 days p.i., the Foxp3^+^CD4 T cell population showing high CD25 and CD122 levels was also reduced in *P2rx7*^-/-^ mice compared with that in B6 mice (S3D and S3E Fig).

Because the P2X7 receptor significantly affected the Th1 and Tfh cell counterbalance during acute *Pc* malaria, we then evaluated the P2X7 expression in B6 CD4 T cells. We also assessed the expression of CD39 ecto-nucleotidase, which can down-regulate P2X7 signaling by degrading eATP [18]. The P2X7 and CD39 levels were increased at 4 days p.i. in T-bet^+^Bcl6^+^ cells but not in T-bet^-^Bcl6^-^ cells (S3F Fig). Remarkably, at 7 days p.i., the P2X7 receptor was mostly expressed in T-bet^lo^Bcl6^hi^ cells, whereas T-bet^hi^Bcl6^lo^ cells exhibited considerably more CD39 (Fig 3D). In fact, two different CD4 T cell populations were identified at 7 days p.i. according to P2X7 and CD39 expression: the P2X7^lo^CD39^hi^ (T-bet^hi^Bcl6^lo^) and P2X7^hi^CD39^lo^ (T-bet^lo^Bcl6^hi^) subsets (Fig 3E).

In summary, P2X7 deficiency impairs the Th1 cell differentiation from the onset of *Pc* infection. This effect was counterbalanced by a percentage increase of T-bet^lo^Bcl6^hi^ CD4 T cells.

### Lack of P2X7 receptor leads to enhanced antibody response during chronic *Pc* malaria

**Fig 4:**
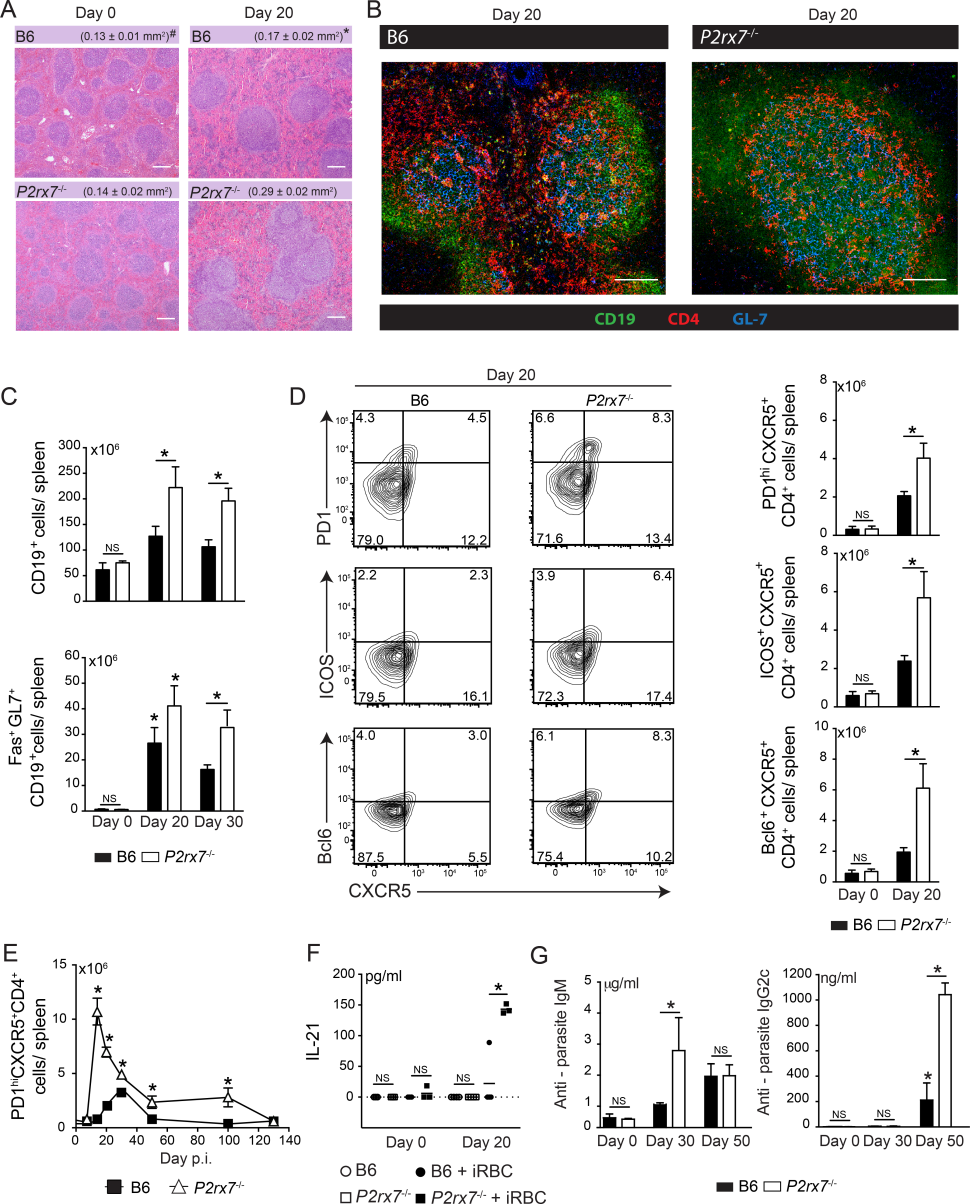
Splenic Tfh cell responses in infected B6 and *P2rx7*^-/-^ mice. (A-G) B6 and *P2rx7*^-/-^ female mice were analyzed at 7,14, 20, 30, 50, 100 and 130 days p.i. with 1 × 10^6^ *Pc*-iRBCs. Naïve mice were used as controls (day 0). The data were expressed as means ± SD (*n* = 3–5) of one representative experiment out of three. Significant differences were observed for the (*) B6 and *P2rx7*^-/-^ groups with *p* < 0.05, using the Mann Whitney U test (NS, not significant). (A) Hematoxilyn-eosin stained sections show splenic follicular hyperplasia in *P2rx7*^-/-^ mice (40x magnification; bar scales correspond to 200 μm). (^#^) The mean areas of lymphoid follicles are shown. (B) Confocal immunofluorescence images (100x magnification; bar scales correspond to 400 μm) of splenic sections are shown. Tissue slices were stained for CD19 (green), CD4 (red) and GL7 (blue). (C) CD19^+^ and Fas^+^GL7^+^CD19^+^ cell numbers per spleen were determined by flow cytometry. (D) Contour plots show PD1, ICOS and Bcl6 *versus* CXCR5 expression in CD4^+^ cells. PD1^+^CXCR5^+^CD4^+^, ICOS^+^CXCR5^+^CD4^+^ and Bcl6^+^CXCR5^+^CD4^+^ cell numbers per spleen are shown in the column bar graphs. (E) PD1^+^CXCR5^+^CD4^+^ cell numbers per spleen were determined by flow cytometry. (F) IL-21 concentrations were determined by ELISA in the supernatants of splenocytes stimulated or not with iRBCs (splenocyte/3 iRBCs). (G) Anti-parasite IgM and IgG2c serum concentrations were determined by ELISA.

The early increase in the proportion of T-bet^lo^Bcl6^hi^ CD4 T cells in infected *P2rx7*^-/-^ mice motivated a careful examination of the development of Tfh cells during chronic *Pc* malaria. First, we observed hyperplasia of splenic secondary lymphoid follicles in the absence of the P2X7 receptor (Fig 4A). Confocal microscopy analysis of these follicles at 20 days p.i. revealed many CD4 T cells in extensive GL7-stained germinal centers (Fig 4B). Consistently, at 20 and 30 days p.i., *P2rx7*^-/-^ mice showed higher B cell numbers per spleen, including germinal center (Fas^+^GL7^+^CD19^+^) B cells (Fig 4C). P2X7 deficiency also led to the development of an increased Tfh cell population that was identified by the expression of inducible T cell co-stimulation (ICOS), CXCR5, PD1 and Bcl6 (Fig 4D). Remarkably, a prominent Tfh cell response occurred at 14 days p.i. in *P2rx7*^-/-^ mice yielding an earlier peak of Tfh cell numbers than in B6 mice; the Tfh cell population remained augmented in *P2rx7*^-/-^ mice until day 100 p.i. (Fig 4E). We also evaluated the IL-21 production, which is a Tfh cell signature and contributes to functional Tfh cell generation in *Pc* malaria [33]. Following stimulation *in vitro* with iRBCs, *P2rx7*^-/-^ splenocytes at 20 days p.i. produced higher amounts of IL-21 than B6 counterparts (Fig 4F). Furthermore, the serum concentrations of anti-parasite IgM at 30 days p.i. and IgG2c at 50 days p.i. were higher in *P2rx7*^-/-^ mice than in B6 mice (Fig 4G). This data suggests that P2X7 signaling controls the Tfh cell population and anti-parasite antibody production during chronic *Pc* infection.

### Th1 cell response is impaired in chronically infected *P2rx7*^-/-^ mice

**Fig 5:**
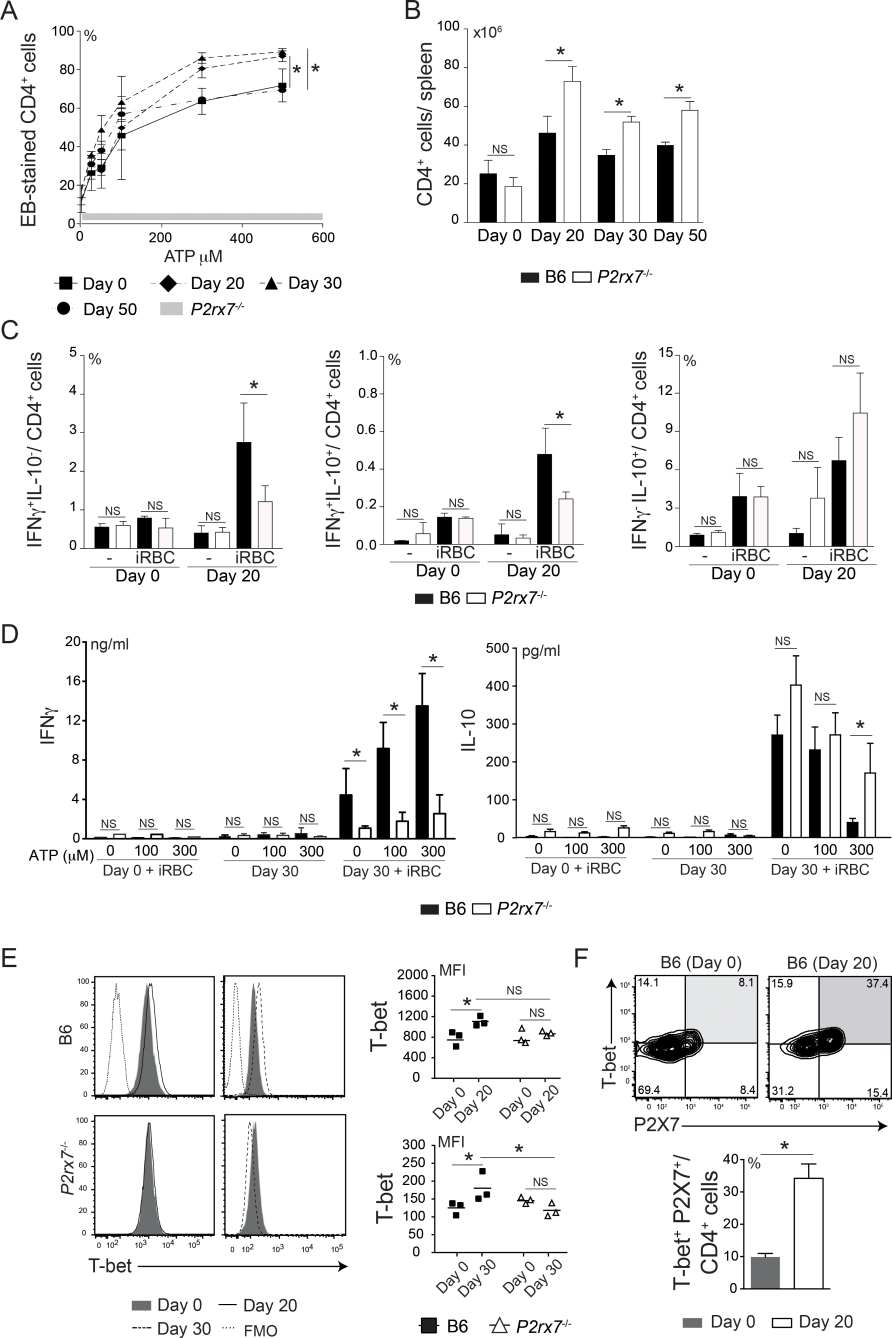
Splenic Th1 cell responses in chronically infected B6 and *P2rx7*^-/-^ mice. (A-F) B6 and *P2rx7*^-/-^ female mice were analyzed at 20, 30 and 50 days p.i. with 1 × 10^6^ *Pc*-iRBCs. Naïve mice were used as controls (day 0). The data were expressed as means ± SD (*n* = 3–5) of one representative experiment out of three. Significant differences were observed for the (*) indicated groups with *p* < 0.05, using the Mann Whitney U test (NS, not significant). (A) Splenocytes were stimulated or not with 25–500 μM ATP. EB-stained CD4^+^ cell percentages were determined by flow cytometry. Horizontal gray bar represents the confidence interval obtained with *P2rx7*^-/-^ cells. (B) CD4^+^ cell numbers per spleen were determined by flow cytometry. (C) Splenocytes were stimulated or not with iRBCs (1 splenocyte/ 3 iRBCs). IFNγ^+^IL-10^-^, IFNγ^+^IL-10^+^ and IFNγ^-^IL-10^+^ cell percentages in CD4^+^ cells were determined by flow cytometry. (D) IFNγ and IL-10 concentrations were determined by ELISA in the supernatants of splenocytes described in E, which were stimulated or not with 100–300 μM ATP. (E) Histograms show T-bet expression in CD4^+^ cells. FMO controls are shown in the histograms. The MFIs of T-bet expression are shown in the scatter plots. (F) Contour plots show T-bet and P2X7 expression in CD4^+^ cells. T-bet^+^P2X7^+^ cell percentages in CD4^+^ cells are shown in the column bar graph.

The higher parasitemias observed in chronically infected *P2rx7*^-/-^ mice compared with those of their B6 counterparts suggested that the P2X7 receptor is required for the development of acquired immunity to *Pc* malaria. Because of the key role of Th1 cells in complete parasite elimination [25]-[27], the splenic CD4 T cell response was also analyzed in chronically infected B6 and *P2rx7*^-/-^ mice. The amounts of additional ATP required to induce P2X7-mediated pore formation in B6 CD4 T cells were still lower at 20 and 30 days p.i. compared to that in non-infected mice, returning to control levels at 50 days p.i. (Fig 5A). Unlike in acute infection, higher CD4 T cell numbers per spleen were observed in *P2rx7*^-/-^ mice than in B6 mice during chronic infection (Fig 5B). The analysis of CD4 T cell subsets, as previously defined [26], revealed larger populations of effector (T_E_), effector memory (T_EM_) and central memory (T_CM_) cells in chronically infected *P2rx7*^-/-^ mice (S4A Fig). Despite these increases, the lack of the P2X7 receptor impaired IFNγ secretion, without affecting IL-10 secretion, as observed during acute *Pc* infection (Fig 5C). Most IFNγ- and IL-10-producing cells exhibited a T_EM_ cell phenotype at 20 days p.i. (S4B Fig). Concordantly, reduced amounts of IFNγ, but not of IL-10, were detected in the supernatants of iRBC-stimulated *P2rx7*^-/-^ splenocytes at 30 days p.i. (Fig 5D). Furthermore, ATP stimulation of B6 splenocytes boosted P2X7-dependent IFNγ production and inhibited P2X7-independent IL-10 production.

Subsequently, we investigated whether the P2X7 receptor is required to induce and/or maintain T-bet expression in splenic CD4 T cells from chronically infected mice. CD4 T cells from B6 mice maintained high expression of this transcription factor at 20 and 30 days p.i. while *P2rx7*^-/-^ CD4 T cells did not (Fig 5E). Furthermore, B6 CD4 T cells at 20 days p.i. co-expressed T-bet and P2X7 receptor (Fig 5F). At 30 days p.i., T-bet up-regulation was observed in B6 CD4 T_E/EM_ and T_CM_ cells but not in *P2rx7*^-/-^ counterparts (S5A Fig). The B6 T_E/EM_ and T_CM_ cells also exhibited high P2X7 levels at day 20 p.i. (S5B Fig). These results indicate that P2X7 receptor is required for the Th1 cell response during chronic *Pc* malaria.

### P2X7 receptor mediates Tfh cell death during chronic *Pc* malaria

**Fig 6:**
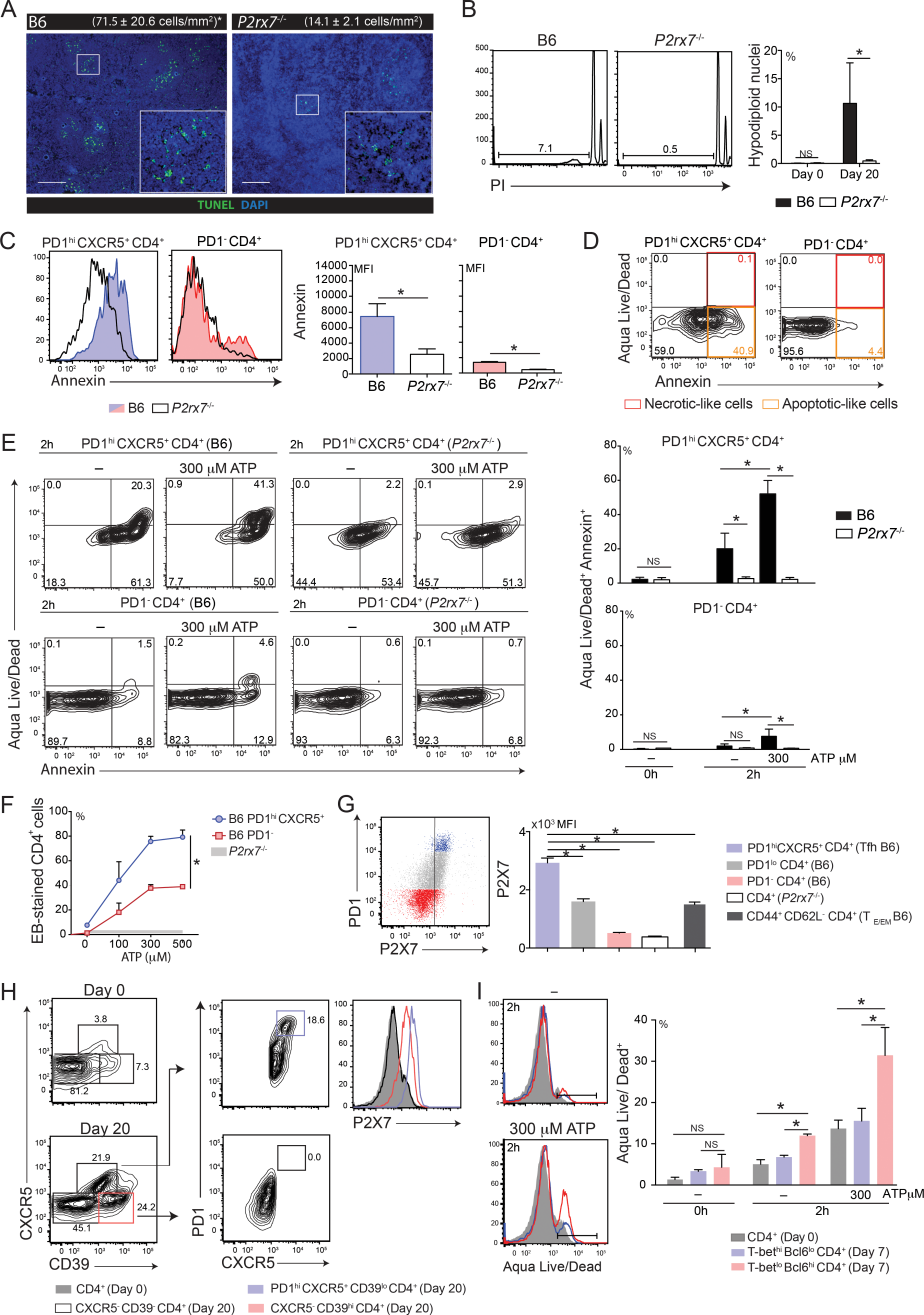
P2X7-mediated Tfh cell death and ATP sensitivity in infected B6 and *P2rx7*^-/-^ mice. (A-I) B6 and *P2rx7*^-/-^ female mice were analyzed at 7 and 20 days p.i. with 1 × 10^6^ *Pc*-iRBCs. Naïve mice were used as controls (day 0). The data were expressed as means ± SD (*n* = 3–5) of one representative experiment out of three. Significant differences were observed for the (*) indicated groups with *p* < 0.05, using (B, C, E and F) the Mann Whitney U test and (G and I) the Kruskal-Wallis test (NS, not significant). (A) Immunofluorescence images (40x magnification; bar scales correspond to 200 μm) of splenic sections were obtained using TUNEL assay. (B) Histograms show propidium iodide (PI) incorporation in CD4^+^ cells. Hypodiploid nucleus percentages in CD4^+^ cells are shown in the column bar graph. (C) Histograms show annexin V staining in PD1^hi^CXCR5^+^CD4^+^ and PD1^-^CD4^+^cells. The MFIs of annexin V staining are shown in the column bar graphs. (D) Contour plots show PD1^hi^CXCR5^+^CD4^+^ and PD1^-^CD4^+^ cells, which were stained for annexin V and Aqua Live/Dead reagent. (E) Contour plots show PD1^hi^CXCR5^+^CD4^+^ and PD1^-^CD4^+^ cells, which were stained for annexin V and Aqua Live/Dead reagent. Cells were cultured for 2 h with or without 300 μM ATP. (F) Splenocytes were stimulated or not with 100–500 μM ATP. Histograms show EB-stained PD1^hi^CXCR5^+^CD4^+^ and PD1^-^CD4^+^ cells. EB-stained CD4^+^ cell percentages are shown in the line graph. Horizontal gray bar represents the confidence interval obtained with *P2rx7*^-/-^ cells. (G) Dot plot shows PD1 and P2X7 expression in CD4^+^ cells. The MFIs of P2X7 expression in PD1^hi^CXCR5^+^CD4^+^, PD1^lo^CD4^+^, PD1^-^CD4^+^, CD4^+^ and CD44^+^CD62L^-^CD4^+^ cells are shown in the column bar graph. (H) Contour plots show CXCR5 and CD39 expression in CD4^+^ cells, and PD1 and CXCR5 expression in CXCR5^+^CD39^lo^CD4^+^ or CXCR5^-^CD39^hi^CD4^+^. Histograms show P2X7 expression in PD1^hi^CXCR5^+^CD39^lo^CD4^+^, CXCR5^-^CD39^hi^CD4^+^, CXCR5^-^CD39^-^CD4^+^ and CD4^+^ cells. (I) Histograms show Aqua Live/Dead staining in CD4^+^, T-bet^hi^Bcl6^lo^CD4^+^ and T-bet^lo^Bcl6^hi^CD4^+^ cells. Aqua Live/Dead^+^ cell percentages are shown in the column bar graph.

One explanation for our previous results is that P2X7 signaling promotes Th1 cell differentiation to the detriment of Tfh cell differentiation during blood-stage *Pc* malaria by inducing the expression of T-bet, rather than Bcl6. Alternatively, but not mutually exclusive, P2X7 signaling could mediate splenic Tfh cell death as described for Peyer’s patches [16]. Therefore, we examined whether splenic Tfh cells at day 20 p.i. were susceptible to ATP-induced cell death. Using the terminal deoxynucleotidyl transferase dUTP nick end labeling (TUNEL) assay [40], we identified apoptotic cells mostly in the spleen of B6 mice than of *P2rx7*^-/-^ mice at 20 days p.i. (Fig 6A). Aggregates of TUNEL^+^ cells were seen in the lymphoid follicles. Furthermore, at 20 days p.i., lower percentages of hypodiploid nuclei were detected in *P2rx7*^-/-^ CD4 T cells than in B6 CD4 T cells (Fig 6B). Because phosphatidylserine (PS) exposure precedes P2X7-mediated T cell death [15], annexin V staining was also compared in Tfh and non-Tfh cells. In infected B6 mice, most Tfh cells were labeled with annexin V, whereas non-Tfh cells were mainly negative (Fig 6C). Lower annexin V staining was observed in *P2rx7*^-/-^ Tfh cells than in B6 Tfh cells, but Tfh cells still presented higher labeling than non-Tfh cells in the absence of P2X7 receptor. Based on the results of Aqua Live/Dead staining, most annexin V^+^ Tfh cells maintained membrane integrity (Fig 6D). However, after 2 h in culture, a large proportion of B6 Tfh cells died spontaneously as evidenced by the co-expression of annexin V and Aqua Live/Dead staining (Fig 6E). This process was mediated by the P2X7 receptor and increased in the presence of 300 μM ATP. In contrast, most non-Tfh cells remained alive even following ATP stimulation. Additionally, ATP-induced P2X7-mediated pore formation was observed mostly in Tfh cells compared with non-Tfh cells (Fig 6F).

In order to determine the mechanism responsible for the greater sensitivity of Tfh cells to eATP, we evaluated P2X7 and CD39 expression in Tfh and non-Tfh cells from chronically infected B6 mice. At 20 days p.i., Tfh cells exhibited higher levels of P2X7 receptor than non-Tfh cells and T_E/EM_ cells (Fig 6G), which contained both non-Tfh cells and Tfh cells (S5C Fig). Of note, a direct correlation between P2X7 and PD1 expression seems to occur in B6 CD4 T cells. Furthermore, two distinct CD39^+^ CD4 T cell subsets were identified in chronically infected B6 mice; PD1^hi^CXCR5^+^CD39^lo^ (Tfh) cells expressed more P2X7 receptor than CXCR5^-^CD39^hi^ (non-Tfh) cells (Fig 6H).

Because T-bet^lo^Bcl6^hi^ cells at day 7 p.i. also expressed a P2X7^hi^CD39^lo^ phenotype (Fig 3G), we wondered if the early stages of Tfh cell differentiation were also susceptible to ATP-induced cell death. In fact, a higher proportion of T-bet^lo^Bcl6^hi^ cells died spontaneously after 2 h in culture compared with that of T-bet^hi^Bcl6^lo^ cells, a phenomenon that was also observed in the presence of 300 μM ATP. (Fig 6I).

These results indicate that P2X7-mediated cell death contributes to control the Tfh cell response during chronic *Pc* malaria. The high expression of the P2X7 receptor concomitantly with low CD39 levels explains the great sensitivity of the Tfh cell lineage to eATP.

### The P2X7 deficiency in CD4 T cells impairs Th1 cell differentiation and increases the Tfh cell population

**Fig 7:**
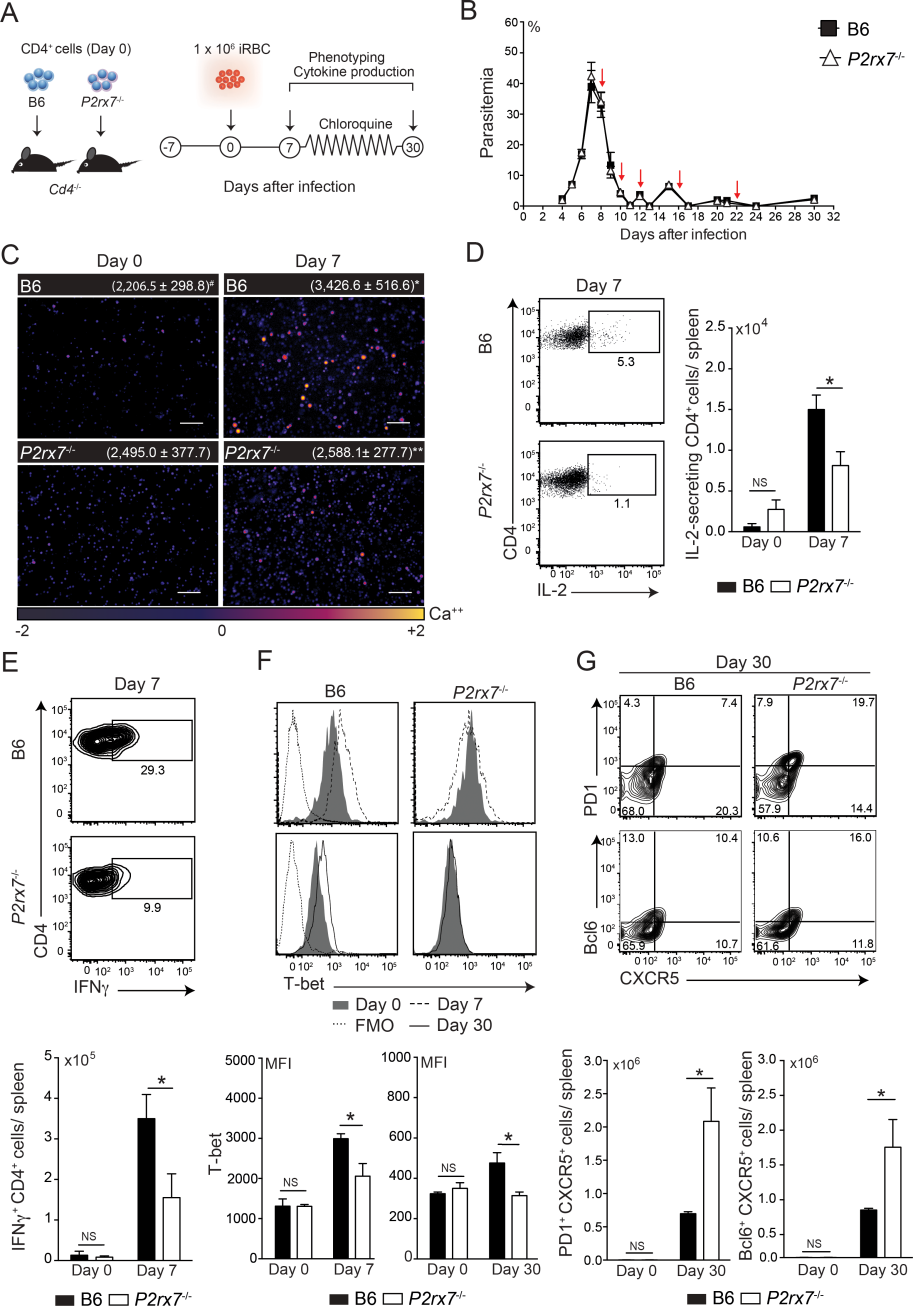
Splenic Th1 and Tfh cell differentiation in *Cd4*^-/-^ mice adoptively transferred with B6 or *P2rx7*^-/-^ CD4 cells and infected with *Pc* parasites. (A-G) Naïve CD4^+^ cells from B6 and *P2rx7*^-/-^ female mice were transferred into *Cd4*^-/-^ female mice that were infected with 1 × 10^6^ *Pc*-iRBCs 7 days later. Splenic CD4^+^ cells were analyzed at 7 and 30 days p.i. Non-infected mice were used as controls (day 0). The data were expressed as means ± SD (*n* = 3–5) of one representative experiment out of three. Significant differences were observed for the (*) B6 and *P2rx7*^-/-^ groups with *p* < 0.05, using the Mann Whitney U test (NS, not significant). (A) A schematic illustration of the experimental protocol is shown. (B) Parasitemia curves are shown. Arrows indicate the days of chloroquine treatment. (C) Immunofluorescence images (100x magnification bar scales correspond to 100 μm) showing cytosolic calcium levels in purified CD4^+^ cells were obtained using the calcium flux assay. (^#^) The mean corrected total cell fluorescence (CTCF) of CD4^+^ cells are shown. (D) Dot plots show IL-2-secreting CD4^+^ cells. IL-2-secreting CD4^+^ cell numbers per spleen are shown in the column bar graph. (E) Contour plots show intracellular IFNγ-stained CD4^+^ cells. IFNγ^+^CD4^+^ cell numbers per spleen are shown in the column bar graph. (F) Histograms show T-bet expression in CD4^+^ cells. FMO controls are shown in the histograms. The MFIs of T-bet expression are shown in the column bar graphs. (G) Contour plots show PD1 and Bcl6 *versus* CXCR5 expression in CD4^+^ cells. PD1^+^CXCR5^+^CD4^+^ and Bcl6^+^CXCR5^+^ cell percentages in CD4^+^ cells are shown in the column bar graphs.

To verify whether P2X7 expression in CD4 T cells is required for Th1 cell differentiation during *Pc* malaria, splenic CD4 T cells were sorted from naïve B6 or *P2rx7*^-/-^ donors and transferred into *Cd4*^-/-^ recipients that were infected with *Pc* parasites a week later (Fig 7A). The treatment with subcurative doses of chloroquine controlled parasitemias at comparable levels in both mouse groups (Fig 7B). A similar increase in CD4 T cell numbers per spleen was observed in these mice at 30 days p.i. (S6A Fig). Nevertheless, *P2rx7*^-/-^ CD4 T cells generated lower numbers of T_EM_ and T_CM_ cells than B6 CD4 T cells (S6B Fig). Demonstrating the role of P2X7 signaling for the early CD4 T cell activation during acute *Pc* malaria, higher cytosolic calcium levels were observed at 7 days p.i. in B6 CD4 T cells compared with *P2rx7*^-/-^ CD4 T cells (Fig 7C). Impaired IL-2 and IFNγ production accompanied the lower cytosolic calcium levels in *P2rx7*^-/-^ CD4 T cells (Fig 7D and 7E). In line with these findings, at 7 and 30 days p.i., T-bet up-regulation was observed in B6 CD4 T cells but not in *P2rx7*^-/-^ CD4 T cells (Fig 7F). Supporting the concept that P2X7 signaling changes the Th1/Tfh cell balance during *Pc* malaria, the Tfh cell population was significantly increased when CD4 T cells lacked the P2X7 receptor (Fig 7G).

**Fig 8:**
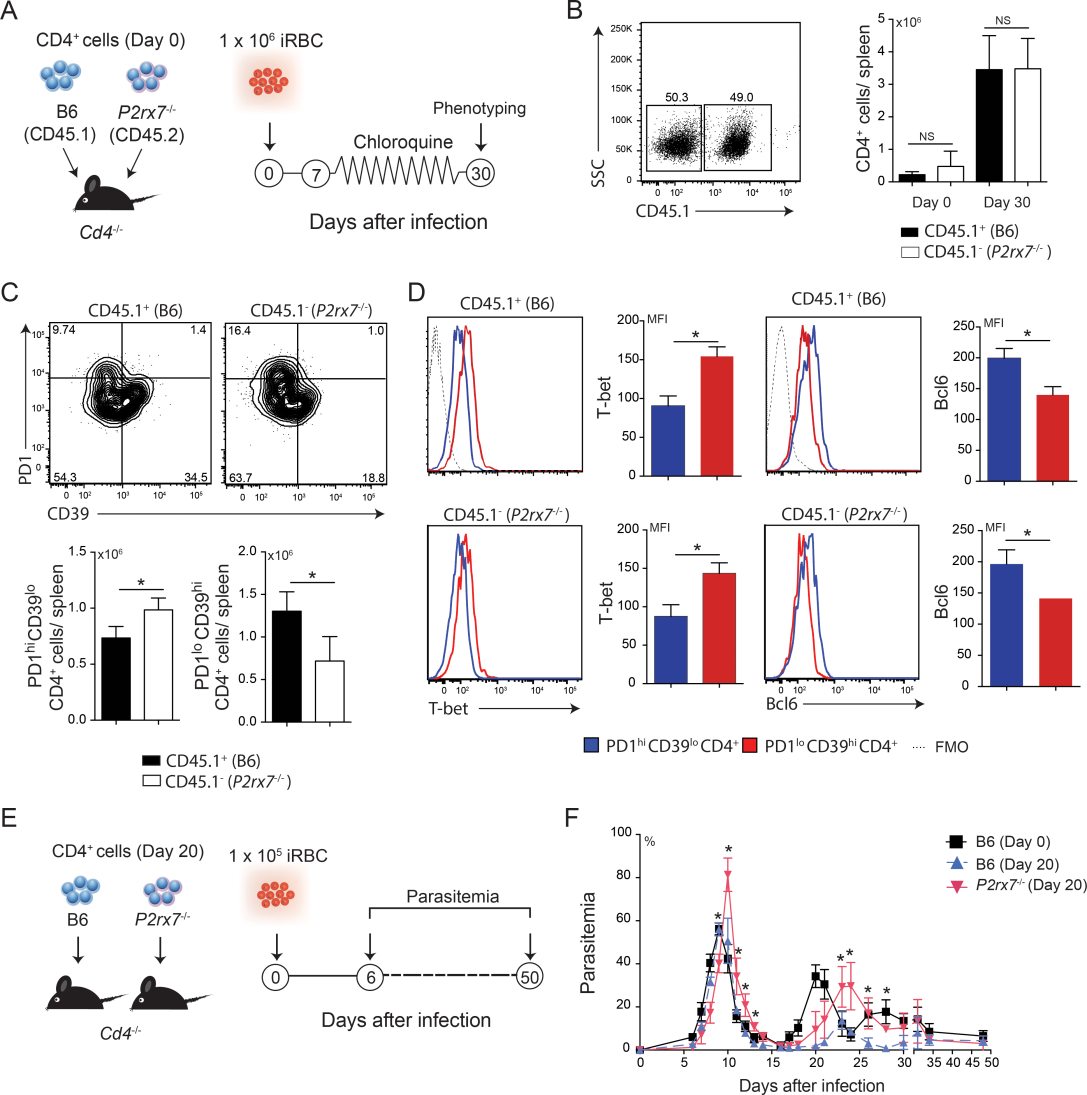
Splenic B6 or *P2rx7*^-/-^ CD4 cell co-transfer and protection against *Pc* infection. (A-D) Naïve CD4^+^ cells from B6 (CD45.1) and *P2rx7*^-/-^ (CD45.2) female mice were co-transferred into *Cd4*^-/-^ female mice that were then infected with 1 × 10^6^ *Pc*-iRBCs. Splenic CD4^+^ cells were analyzed at 30 days p.i. Non-infected mice were used as controls (day 0). The data were expressed as means ± SD (*n* = 5) of one representative experiment out of three. Significant differences were for the (*) indicated groups with *p* < 0.05, using the Mann Whitney U test (NS, not significant). (A) A schematic illustration of the experimental protocol is shown. (B) Dot plot shows CD45.1^+^CD4^+^ and CD45.1^-^CD4^+^ cells at 30 days p.i. CD45.1^+^CD4^+^ and CD45.1^-^CD4^+^ cell numbers per spleen are shown in the column bar graph. (C) Contour plots show PD1 and CD39 expression in CD4^+^ cells. PD1^hi^CD39^lo^CD4^+^ and PD1^lo^CD39^hi^CD4^+^ cell percentages are shown in the column bar graphs. (D) Histograms show T-bet and Bcl6 expression in PD1^hi^CD39^lo^CD45.1^+^CD4^+^, PD1^lo^CD39^hi^CD45.1^+^CD4^+^, PD1^hi^CD39^lo^CD45.1^-^CD4^+^ and PD1^lo^CD39^hi^CD45.1^-^CD4^+^cells. FMO controls are shown in the histograms. The MFIs of T-bet and Bcl6 expression are shown in the column bar graphs. (E-F) CD4^+^ cells from B6 and *P2rx7*^-/-^ female mice at 20 days p.i. were transferred into *Cd4*^-/-^ female mice that were infected with 1 × 10^5^ *Pc*-iRBCs. *Cd4*^-/-^ mice transferred with naïve B6 cells were used as controls. The data were expressed as means ± SD (*n* = 4–6) of one representative experiment out of three. Significant differences were observed for the (*) mice transferred with B6 cells at 0 and 20 days p.i. and (**) mice transferred with B6 and *P2rx7*^-/-^ cells at 20 days p.i. with *p* < 0.05, using the Mann Whitney U test. (E) A schematic illustration of the experimental protocol is shown. (F) Parasitemia curves are shown.

Next, we examined how B6 and *P2rx7*^-/-^ CD4 T cells differentiate in the same environment after *Pc* infection. Therefore, *Cd4*^-/-^ mice co-transferred with B6 (CD45.1) and *P2rx7*^-/-^ (CD45.2) CD4 T cells were infected with *Pc* parasites a week later (Fig 8A). At 30 days p.i., similar numbers of B6 and *P2rx7*^-/-^ CD4 T cells were observed in the spleen (Fig 8B). Consistent with our previous results, the proportion of PD1^lo^CD39^hi^ CD4 T cells was greater in the B6 population, whereas PD1^hi^CD39^lo^ CD4 T cells predominated in the *P2rx7*^-/-^ population (Fig 8C). For the B6 and *P2rx7*^-/-^ populations, PD1^lo^CD39^hi^ and PD1^hi^CD39^lo^ CD4 T cell subsets showed higher T-bet and Bcl6 expression, respectively (Fig 8D). In conclusion, P2X7 expression in CD4 T cells is sufficient to promote Th1 cell differentiation over Tfh cells during *Pc* malaria.

### The balanced Th1/Tfh cell population generated from B6 CD4 T cells confers better protection against *Pc* malaria

Our previous results indicate that P2X7 expression in CD4 T cells is critical for generating a balanced Th1/Tfh cell population during chronic *Pc* malaria, while an increased Tfh cell population develops in the absence of P2X7 receptor. To assess the consequences of that for protective immunity, splenic CD4 T cells from B6 or *P2rx7*^-/-^ mice at day 20 p.i. were transferred into *Cd4*^-/-^ recipients that were infected with *Pc* parasites (Fig 8E). *Cd4*^-/-^ mice transferred with naïve B6 CD4 T cells were used as controls. Significantly lower first and second parasitemia peaks were observed in *Cd4*^-/-^ mice transferred with B6 CD4 T cells at day 20 p.i. compared with those transferred with *P2rx7*^-/-^ CD4 T cells at day 20 p.i. (Fig 8F). Notably, *Cd4*^-/-^ mice transferred with naïve B6 CD4 T cells or *P2rx7*^-/-^ CD4 T cells at day 20 p.i. failed to control recrudescent parasitemia up to a month of infection. We concluded that the balanced Th1/Tfh cell population that develops in infected B6 mice is more efficient in controlling the first and second parasitemia peaks than the increased Tfh cell population generated in infected *P2rx7*^-/-^ mice

## Discussion

The P2X7 receptor has been implicated in both the protection and exacerbation of infectious diseases by inducing the activation and death of infected macrophages [41–43]. An important concept that emerges from our study is that eATP recognition by P2X7 receptor promotes the differentiation of Th1 cells to the detriment of Tfh cells during *Pc* malaria and thus contributes to protection. The first evidence in this direction was the susceptibility pattern of *P2rx7*^-/-^ mice that closely resembled the one previously described for IFNγ deficiency, in which most males died during acute *Pc* infection and females developed increased acute and chronic parasitemia [23]. The immunosuppressive effects of testosterone in the production of and response to cytokines were implicated in the higher susceptibility of male IFNγ^-/-^ mice to *Pc* parasites, a phenomenon that was recapitulated in *P2rx7*^-/-^ males due to their extremely low Th1 response.

The eATP concentrations following iRBC rupture achieved, at least in some microenvironments, the threshold required for P2X7 activation in CD4 T cells, as observed for other pathological processes [44], [45]. Furthermore, splenic CD4 T cells became highly responsive to eATP at the beginning of *Pc* infection, which may have contributed to amplifying the early lymphocyte response. Indeed, the P2X7 receptor was important for CD4 T cell proliferation and IFNγ production during acute *Pc* malaria and consequently for IgM and IgG2c secretion that depends, at least partially, on CD4 T cell help [24]. Both IFNγ and low-affinity antibodies participate in the control of acute *Pc* malaria [22], [23], [46]. The P2X7 expression was also critical for IFNγ production during chronic disease. This finding corroborates our previous studies suggesting that a Th1 response is required, together with anti-parasite antibodies, to resolve a persistent *Plasmodium* infection [25], [27]. Moreover, the inflammatory response may be further potentiated during *Pc* infection by the inhibitory effect of eATP on IL-10 secretion, which was shown here to be independent of the P2X7 receptor.

In addition to the important contribution of the P2X7 receptor in boosting the CD4 T cell response during acute *Pc* malaria, P2X7 signaling was critical for driving Th1/Tfh cell differentiation. On day 4 p.i., CD4 T cells had apparently not yet been committed on Th1 and Tfh cell lineages because there was only a small population expressing both T-bet and Bcl6. However, the two subsets could clearly be distinguished at 7 days p.i. by the preferential expression of T-bet, CD25, CD122 and CD39 in Th1-biased cells and Bcl6 and P2X7 receptor in Tfh-biased cells; P2X7 deficiency reduced the proportion of T-bet^hi^Bcl6^lo^ cells and augmented that of T-bet^lo^Bcl6^hi^ cells. In line with these findings, a single-cell RNA seq analysis of Th1/Tfh cell differentiation during acute *Pc* malaria showed that these subsets emerge in parallel by day 7 p.i.; *Entpd1* (CD39) and *P2rx7* expression was identified as Th1 and Tfh signature, respectively [47]. To explain our data showing low antibody production in acutely infected *P2rx7*^-/-^ mice, we considered the possibility that T-bet^hi^Bcl6^lo^ and T-bet^lo^Bcl6^hi^ cell subsets could provide help for B cells at the early *Pc* infection; P2X7 deficiency reduced only the T-bet^hi^Bcl6^lo^ cell population. Supporting this idea, both subsets displayed an early Tfh phenotype expressing CXCR5 and PD1 at levels below those of fully differentiated Tfh cells.

During chronic *Pc* malaria, there was a sharp expansion of the Tfh cell population in *P2rx7*^-/-^ mice that resulted in higher serum levels of anti-parasite IgM and IgG2c than in the B6 counterparts. The persistence of Th1 bias in chronically infected B6 mice was evidenced by T-bet and P2X7 co-expression in T_E/EM_ and T_CM_ cells; however, CD4 T cells from *P2rx7*^-/-^ mice did not express this transcription factor. Of note, the selective absence of the P2X7 receptor in CD4 T cells was sufficient to change the Th1/Tfh cell balance. This effect was shown in mice transferred with either *P2rx7*^-/-^ or B6 CD4 T cells in which parasitemia was maintained at similar levels by drug treatment, as well as in mice co-transferred with both CD4 T populations. In both experimental conditions, there was no apparent competitive advantage between the *P2rx7*^-/-^ and B6 CD4 T cell populations that displayed similar sizes a month after infection. P2X7 deficiency in CD4 T cells resulted in a lower increase in the T_E_, T_EM_ and T_CM_ cell populations a month after infection. The opposing effect obtained in chronically infected *P2rx7*^-/-^ mice may be a consequence of higher parasitemia found in these animals in relation to chronically infected B6 mice.

The P2X7 signaling can influence Th1/Tfh cell differentiation by inducing the T-bet-controlled Th1 cell program, which hinders the development of the Bcl6-controlled Tfh cell program. In fact, P2X7 deficiency led to lower expression of Blimp-1 in CD4 T cells and this transcription factor is a known antagonist of Bcl6 [37]. It is generally accepted that P2X7 signaling in T cells amplifies T cell receptor (TCR)-induced calcium influx and thus increases IL-2 secretion [48], [49]. Accordingly, calcium influx and IL-2 secretion were dependent on P2X7 expression in CD4 T cells from acute *Pc* malaria. Furthermore, it has been shown that eATP is required for IL-2 and IFNγ secretion by antigen-specific T cells [50]. IL-2 induces the signal transducer and activator of transcription 5 (STAT5) in CD4 T cells that up-regulates the IL-12 receptor β2-chain, T-bet and Blimp-1 [51], [52]. Moreover, IL-2-mediated activation of the mammalian target of rapamycin complex 1 (mTORc1) kinase axis up-regulates Blimp-1 expression and shifts differentiation away from Tfh cells, instead promoting that of Th1 cells [53]. Another evidence that IL-2 induced by P2X7 signaling influences the CD4 T cell response to *Pc* malaria was the lower increase of CD4 T cells expressing IL-2 receptor α- and β-chains in infected *P2rx7*^-/-^ mice compared with the B6 counterparts. Other cytokines can induce T-bet expression, such as IL-12, IL-27 and IFNα [38], [54], but the relationship of these signaling pathways with P2X7 receptor is unclear.

An alternative non-exclusive molecular mechanism by which the P2X7 receptor can change the Th1/Tfh balance relies on the high susceptibility of Tfh cells to ATP-induced cell death. Suggesting that this mechanism operates during *Pc* infection, P2X7 deficiency reduced the apoptotic cell death in germinal centers and PS exposure in Tfh cells. Furthermore, Tfh cells are particularly prone to die spontaneously through the P2X7 receptor. The relatively low CD39 expression may be insufficient to degrade eATP rapidly and thus prevent ATP-induced Tfh cell death, which can be accelerated by the extremely high levels of the P2X7 receptor in these cells. Similarly, in mouse Peyer’s patches and human tonsils, the *P2rx7* gene is highly expressed in Tfh cells that are particularly responsive to eATP and undergo P2X7-mediated cell death [16]. In this study, as in ours, Tfh cell death was evaluated in B6 mice, which have an allelic mutation in the predicted death domain of P2X7 receptor that reduces eATP sensitivity [55]. Macrophages and lymphocytes from B6 mice respond to ATP stimulation and undergo P2X7-mediated cell death [16], [43], [56], although they are more resistant than cells from other mouse strains [55]. Therefore, the effects of eATP are expected to be greater on Tfh cells expressing the unmutated *P2rx7* gene. Regarding how splenic Tfh cells come into contact with eATP during *Pc* malaria, it is likely that the absence of compartmentalization between the white and red pulp observed at the acute infection makes available large amounts of locally released ATP from lysed iRBCs [57]. Lymphocytes stressed or dying as a result of the affinity maturation process, as well as lysed iRBCs in the marginal zone and in the vessels that irrigate the germinal centers, can be the sources of eATP during chronic *Pc* malaria.

The detailed analysis of the P2X7 receptor's role in the immune response to *Pc* infection allowed us to reveal the importance of this molecule in the fine-tuning between Th1 and Tfh cell populations. It is generally accepted that antibody affinity maturation and memory B cells develop properly in *Pc* malaria [58], [59]. Additionally, functional Tfh cells are necessary for an efficient antibody response and resolution of chronic *Pc* parasitemia [33]. Nevertheless, some control of Tfh cell differentiation through the P2X7 receptor appears to be required to generate the Th1 response to *Pc* infection, thus improving the disease outcome. Supporting this idea, mice transferred with B6 CD4 T cells at 20 days p.i. containing both Th1 and Tfh cells showed a better control of the first and second parasitemia peaks than those transferred with the *P2rx7*^-/-^ counterparts where there is a marked predominance of the Tfh phenotype. Of note, P2X7 deficiency led to better control of *P*. *yoelii* 17XNL malaria whose outcome seems to depend more on anti-parasite antibodies and less on IFNγ than in *Pc* malaria [32], [35]. A feasible explanation for this finding is that *Pc* merozoites, when released, rapidly infect nearby erythrocytes, but free *P*. *yoelii* 17XNL parasites are required to remain longer in the extracellular environment to find few reticulocytes, thereby making them easy targets for antibodies. In apparent contradiction to our results, the expansion of the Tfh cell population by inhibiting PD1 and lymphocyte activation gene (LAG)-3, or in mice deficient in IFNα receptor 1, improves the control of both *Pc* and *P*. *yoelii* 17XNL infections [32], [34]. However, the effect of Th1/Tfh imbalance cannot be appreciated under these conditions because IFNγ production is also increased. We propose here that elevated eATP in the spleen, which may occur during *Plasmodium* infection, might modify the CD4 T cell balance toward a more potent inflammatory response and hinder the antibody response. This concept is in line with a recent study showing that the extremely intense inflammatory response during severe *Plasmodium berghei* ANKA infection drastically inhibits Tfh cell differentiation [60]. Thus, our study goes a step further in understanding malaria pathogenesis, suggesting that continuous ATP release and P2X7 signaling might not only promote the Th1 response but also delay antibody production during *Plasmodium* infection.

Our study provides mechanistic insights into malaria pathogenesis by demonstrating the importance of damage signals for the outcome of the disease. By promoting a balanced Th1/Tfh cell response, the immune system becomes more effective in protecting against *Pc* parasites whose control is based on both IFNγ and anti-parasite antibodies. It remains unclear whether the development of a robust Tfh cell response as a result of P2X7 deficiency is responsible for increasing the resistance to *P*. *yoelii* 17XNL infection. Human CD4 T cells express P2X7 receptor and secrete IL-2 following eATP stimulation [48], but it is still unknown whether P2X7 signaling influences the CD4 T cell response in malaria patients. Supporting the role of P2X7 signaling in controlling Tfh cell numbers in humans, tuberculosis patients carrying a single loss-of-function *P2rx7* gene polymorphism produce more IgG against mycobacteria than control groups [61]. This knowledge raises the possibility that the ATP-P2X7 axis can be manipulated with P2X7 agonists and antagonists to change the Th1/Tfh cell balance, aiming to ameliorate pathological conditions or to improve immunization protocols.

## Materials and methods

### Mice, parasites and infection

Six-to-eight-week-old C57BL/6 (B6), B6.SJL-*Ptprc*^*a*^ *Pepc*^*b*^/BoyJ (CD45.1^+/+^), B6.129P2-*P2rx7* ^*tm1Gab*^/J (*P2rx7*^-/-^), B6.Cg-*Fosn1*^*nu*^/J (nude) and B6.129S2-*Cd4*^*tm1Mak*^/J (*Cd4*^-/-^) female and male mice (originally from The Jackson Laboratory) were bred under specific pathogen-free conditions at the Isogenic Mice Facility (ICB-USP, Brazil). The *P2rx7*^-/-^ mice were generated by Pfizer Inc. (USA); a single nucleotide polymorphism panel analysis throughout the genome suggested a B6 genetic background. The experiments were performed in female mice with the exception that females and males were compared. *Pc* and *P*. *yoelii* 17XNL parasites were maintained as described elsewhere [62]. Because the *Pc* schizogonic cycle depends on the host circadian rhythm, the mice were maintained under an inverted light/dark cycle for at least 15 days before infection to access the period adjacent to erythrocyte invasion [63]. Mice were infected intraperitoneally with 1 × 10^6^ iRBCs.

### Ethics statement

All experimental procedures were in accordance with national regulations of ethical guidelines for mouse experimentation and welfare of the Health National Council and Animal Experimentation Brazilian College (COBEA)—Brazil, the protocols being approved by the Health Animal Committee of USP, with permit numbers 050/2009 and 175/2011.

### Parasitemias and clinical analysis

Parasitemias were monitored by microscopic examination of Giemsa-stained blood smears. Body weight variation was determined with respect to the day 0 weight with an analytical balance (Sartorious, USA). Axial temperature was assessed with a digital thermometer (Kent Scientific Co., USA). Hemoglobin serum concentration was evaluated with a hemoglobin kit (Doles Inc., Brazil).

### Cell suspensions

Blood and spleen cells were washed and maintained in cold RPMI 1640 supplemented with penicillin (100 U/ml), streptomycin (100 μg/ml), 2-mercaptoethanol (50 μM), L-glutamine (2 mM), sodium pyruvate (1 mM) and 3% heat-inactivated fetal calf serum. All supplements were purchased from Life Technologies (USA). Leukocytes were obtained in 70% Percoll gradient (GE Health Care, USA). For the cell proliferation assay, spleen CD4 T cells were magnetically purified by negative selection. Non-CD4 T cells were labeled with biotinylated antibodies and streptavidin-coated magnetic particles and then were separated using an EasySep magnet (Stem Cell Technologies, Canada). For the calcium flux assay, spleen CD4 T cells were magnetically purified (LS columns) by positive selection using anti-CD4 microbeads with autoMACS (Miltenyi Biotec, Germany). For adoptive transfer, spleen CD4 T cells were magnetically purified (LS columns) by negative selection using anti-CD19, -IA^b^ and -CD8 microbeads with autoMACS (Miltenyi Biotec, Germany) and then were sorted using a FACS Aria device (BD Biosciences, USA). In some experiments, spleen CD4 T cells were magnetically purified by negative selection using an EasySep magnet and then were sorted using a FACS Aria device.

### Cell phenotyping

Cells (1 × 10^6^) were stained with FITC-, PE-, APC-, PerCP-, PECy7-, APC Cy7-, Pacific Blue-, or AmCyan-labeled monoclonal antibodies (mAbs) (BD Biosciences) to CD4 (H129.19 or GK1.5), CD19 (ID3), CD25 (PC61), CD39 (24DMS1), CD44 (IM7), CD62L (MEL-14), CD122 (TM-β1), CD178 (ICOS) (7E.17G9), CD127 (A7R34), GL7 (GL7), PD1 (J43), CXCR5 (2G8) and P2X7 (1F11). For the detection of intracellular staining, PE-labeled mAb to Bcl-6 (K112-91, BD Biosciences), PerCP-labeled mAb to T-bet (eBio4B10; eBioscience, USA), PE-labeled mAb to Blimp-1 (6D3; BD Biosciences) and APC-labeled mAb to Foxp3 (FJK-16s; eBioscience) were used according to the manufacturer’s instructions. PE-labeled rat IgG1 (BD Bioscience) and PerCP-labeled anti-CD45.1 mAb (A20; BD Bioscience) were used as isotype controls. Annexin V staining was performed in the appropriate binding buffer (10 mM HEPES, 150 mM NaCl, 5 mM KCl, 1 mM MgCl_2_, 1.8 mM CaCl_2_ [pH 7.4]). Cells were analyzed by flow cytometry using a FACSCanto device with DIVA software (BD Biosciences). Data were analyzed with FlowJo software v.7.2.2 (Tree Star Inc., USA).

### Cell permeabilization assay

Blood and spleen cells (1 × 10^6^) were stained with APC-labeled anti-CD4 mAb (BD Biosciences). In some experiments, stained cells were pre-warmed (37°C) in phosphate-buffered saline with 3% bovine serum albumin (Sigma-Aldrich) and then incubated with 25–500 μM ATP (Amersham Bioscience, USA), lysed iRBC supernatant or medium alone for 15 min. The non-infected RBCs (nRBCs) and iRBCs (2 × 10^8^) were lysed with 200 μl of lysis buffer (40 mM NH_4_Cl, 4.2 mM Tris [pH 7.4]) for 5 min at 4°C. Spleen cells (1 × 10^6^) were incubated with 200 μl of RBC supernatants diluted 1:5 in cold RPMI with 1% heat-inactivated fetal calf serum (FCS). The fluorescent 2.5 μM EB dye (Sigma-Aldrich) was added, and the samples were immediately analyzed by flow cytometry.

### ATP determination

ATP concentrations were determined using an ATP bioluminescence assay kit (Sigma-Aldrich). Serum (50 μl/well) was mixed 1:1 with the luciferase reagent. The bioluminescence was quantified in a temperature-controlled luminometer (Berthold, USA).

### Cell proliferation assay

Purified CD4 T cells (3 × 10^7^) were incubated for 20 min at 37°C with 5 μM 5,6-carboxyfluorescein succinimidyl ester (CFSE; Molecular Probes, USA) in phosphate-buffered saline (PBS) with 0.1% bovine serum albumin (BSA, Sigma-Aldrich). CD4 T cells (5 × 10^5^) were cultured with iRBCs (4 × 10^6^) or medium alone, in the presence of spleen cells (5 × 10^5^) from nude mice as a source of APCs, for 72 h at 37°C in a 5% CO_2_ atmosphere, stained with PECy7-labeled anti-CD4 mAb and analyzed by flow cytometry. In the experiments using apyrase or BBG, spleen cells (3 x 10^7^) were stained with CFSE as described above. Cells (1 x 10^6^) were cultured with iRBCs (4 x 10^6^) in the presence or absence of apyrase (20 U/ml) or BBG (35 μM) for 72 h at 37°C in a 5% CO_2_ atmosphere, stained with PE-labeled mAb to CD4 and analyzed by flow cytometry.

### Cytokine detection

For intracellular *ex vivo* detection, spleen cells (1 × 10^6^) were cultured with GolgiStop reagent (containing monensin) according to the manufacturer's instructions for 6 h at 37°C in a 5% CO_2_ atmosphere. For intracellular *in vitro* detection, spleen cells (1 × 10^6^) were cultured with iRBCs (4 × 10^6^) or medium alone for 72 h at 37°C in a 5% CO_2_ atmosphere. The GolgiStop reagent was added at the last 6 h of culture according to the manufacturer's instructions. After washing, cells were surface stained with APC- or Pacific Blue-labeled mAbs to CD4. Cells were then fixed with Cytofix/Cytoperm buffer, stained with PE-labeled mAb to IFNγ (XMG-1.2) and APC-labeled mAb to IL-10 (JESS-16E3) diluted in Perm/Wash buffer, and analyzed by flow cytometry. All reagents were purchased from BD Biosciences. The IL-2 cytokine secretion assay was performed according to the manufacturer's instructions (BD Biosciences). This assay uses a bi-functional mAb capable of binding CD45 and IL-2. Cells (2 × 10^6^) were incubated with the bi-functional mAb for 45 min at 37°C in 5% CO_2_ atmosphere. The IL-2 bound to the surface of cells was detected with PE-labeled anti-IL-2 mAb by flow cytometry. For supernatant cytokine detection, spleen cells (1 × 10^6^) were cultured with 3 × 10^6^ iRBCs in the presence or absence of apyrase (20 U/ml), BBG (35 μM) or medium alone for 72 h at 37°C in a 5% CO_2_ atmosphere. Cytokine concentrations were determined using the OptEIA IFNγ kit (BD Biosciences), OptEIA IL-10 kit (BD Biosciences) and mouse IL-21 ELISA (eBioscience).

### Anti-parasite ELISA

The anti-*Pc* IgM, IgG1 and IgG2c serum levels were quantified by ELISA as described elsewhere [64]. Briefly, 96-well flat-bottom microtest plates (Costar, USA) were coated overnight at 4°C with 8 μg/ml of a total *Pc* extract and saturated with 1% BSA for 3 h. After washing, 100 μl of mouse serum samples (diluted from 1/10 to 1/1,280) were added and left overnight at 4°C. Antibody concentrations were determined using Ig standards. The assays were developed by adding goat anti-mouse Ig isotype peroxidase–conjugated antibodies (Southern Biotechnology Associates, USA) for 45 min, followed by the addition of 100 μl of tetramethylbenzi-dine (Invitrogen, USA). Absorbance was measured at 650 nm with an Epoch Microplate Spectrophotometer (BioTek, USA).

### ELISPOT assay

Ig-producing cells were quantified by the ELISPOT assay as described elsewhere [65]. In brief, 96-well flat-bottom microtest plates (Costar) were coated overnight at 4°C with 10 μg/ml of goat-anti-mouse total Ig and saturated with 1% gelatin (Merck, Germany) in PBS for 120 min. Spleen cells (1 × 10^6^ to 5 × 10^2^ cells/well) were cultured for 6 h at 37°C in a 5% CO_2_ atmosphere. The spots were developed by adding goat anti-mouse Ig isotype biotinylated antibodies overnight, followed by the addition of a phosphatase alkaline-avidin conjugate. All antibodies and conjugates were purchased from Southern Biotechnologies Associates. 5-Bromo chloro 3-indolyl phosphate (BCIP; Sigma-Aldrich) diluted in 2-amino 2-methyl 1-propanol (AMP, Merck) was used as a substrate.

### Calcium flux assay

CD4 T cells (1 × 10^7^) were loaded with a mixture of 4 μM Fura-3AM (Molecular Probes, USA) and 0.7 mg/ml of Probenecid (Sigma-Aldrich) at 37°C for 30 min. After washing, cells (1 × 10^5^) were analyzed in a fluorescence microscope (Nikon Inverted Microscope, Japan) to determine the fluorescence intensity. Intracellular calcium was determined by calculating the corrected total cell fluorescence (CTCF). CTCF = integrated density—(area of selected cell × mean fluorescence of background readings).

### TUNEL assay

Spleens were harvested and frozen in Tissue-Tek O.C.T. (Sakura Finetek, USA). In situ DNA fragmentation in 8-μm-thick slices was performed using the DeadEnd Fluorometric TUNEL System according to the manufacturer’s protocol (Promega, USA).

### DNA fragmentation

APC-labeled anti-CD4 mAb stained cells (1 × 10^6^) were fixed with 70% ethanol. After washing, cells were incubated in DNA extraction buffer (0.2 M Na_2_HPO_4_ and 0.1% Triton x-100, Sigma-Aldrich) at 24°C for 5 min, centrifuged and resuspended in a DNA staining solution (20 μg/ml PI, Sigma-Aldrich). RNase (50 μg) (Invitrogen) was then added to each sample, and the cells were incubated at 24°C for 30 min in the dark. PI incorporation was determined by flow cytometry.

### Histology and immunofluorescence analysis

Spleens were fixed in buffered formol for 12 h and paraffin-embedded. Splenic tissue sections (5 mm) were hematoxylin-eosin stained using standard procedures. Spleens were harvested and immediately embedded in Tissue-Tek O.C.T. (Sakura Finetek, Japan) and snap frozen. Slices measuring 8 μm thick were fixed in acetone and blocked with 3% BSA plus Fc Block (1:100) (BD Bioscience) for 1 h at 24°C. Slides were then stained with anti-CD4-Alexa 700 (RM4-5) (eBioscience), CD19-APC (ID3), GL-7-Biotin (GL7) and Streptavidin-FITC (BD Biosciences) for 2 h at 24°C. After washing, slides were stained with 4',6-diamidino-2-phenylindole (DAPI; Molecular Probes) and mounted with Vectashield mounting medium (Vector, USA). Sections were analyzed by confocal microscopy (Zeiss LSM 780, Germany).

### Adoptive transfer

For CD4 T-cell phenotypic analysis, *Cd4*^-/-^ mice adoptively transferred i.v. with purified CD4 T cells (1.5 × 10^6^) from naïve B6 or *P2rx7*^-/-^ mice were treated i.p. from day 7 p.i. with 3 every-other-day doses of chloroquine (10 mg/kg of body weight/day). Alternatively, *Cd4*^-/-^ mice co-transferred i.v. with purified B6 and *P2rx7*^-/-^ CD4 T cells (1 × 10^6^/each population) from naïve mice were treated i.p. from day 7 p.i. with 3 consecutive daily doses of chloroquine (10 mg/kg of body weight/day). For parasitemia analysis, B6 or *P2rx7*^-/-^ CD4 T cells (1.5 × 10^6^) at day 20 p.i. were transferred i.v. to *Cd4*^-/-^ mice.

### Statistical analysis

Statistical analysis was performed by the Mann-Whitney U test to compare two groups. For more than two groups, data were analyzed by Kruskal-Wallis test. Survival curves were analyzed by the log-rank test using the Kaplan-Meier method. GraphPad Prism 6 software was used, in which differences between groups were considered significant when *p* < 0.05 (5%).

## Supporting information

S1 Fig Malaria parasite development and ATP serum levels in acutely infected B6 mice. (A) The percentages of trophozoites, shizonts and ring stages were determined at 5 days p.i. with 1 × 10^6^ iRBCs (*n* = 3). Arrows indicate the time at which blood samples were collected.
(B) B6 and *P2rx7*^-/-^ female mice were analyzed at 4 and 5 days p.i. with 1 × 10^6^ iRBCs. Naïve mice were used as controls (day 0). The data were expressed as means ± SD (*n* = 3) of one representative experiment out of three. Significant differences were observed for the (*) indicated groups with *p* < 0.05, using the Mann Whitney U test. ATP concentrations were determined by bioluminescence in B6 mouse serum before and after iRBC rupture. The blood samples were collected at 9 a.m. (6.5 ± 0.5% iRBC at day 4 p.i. and 10.5 ± 1.5% iRBCs at 5 days p.i.; >95% trophozoites and schizonts) and 2 p.m. (12.0 ± 1.2% iRBC at day 4 p.i. and 25.0 ± 3.7% iRBCs at 5 days p.i.; >95% ring forms).
(TIF)

S2 Fig Effects of apyrase and BBG in splenic B6 CD4 T cell responses to iRBCs. (A-B) B6 mice were analyzed at 4 days p.i. with 1 × 10^6^ *Pc*-iRBCs. The data were expressed as means ± SD (*n* = 3) of one representative experiment out of three. Significant differences were observed for the (*) indicated groups with *p* < 0.05, using the Mann Whitney U test (NS, not significant).
(A) CFSE-stained splenocytes were stimulated with iRBCs (1 splenocyte/ 4 iRBCs) in the presence or not of apyrase. CFSE^lo^CD4^+^ cell percentages are shown in the column bar graph. IFNγ concentrations were determined by ELISA in the culture supernatants.
(B) CFSE-stained splenocytes were stimulated with iRBCs (1 splenocyte/ 4 iRBCs) in the presence or not of BBG. CFSE^lo^CD4^+^ cell percentages are shown in the column bar graph. IFNγ concentrations were determined by ELISA in the culture supernatants.
(TIF)

S3 Fig Phenotypic characterization of splenic CD4 T cells in acutely infected B6 and *P2rx7*^-/-^ mice. (A-F) B6 and *P2rx7*^-/-^ female mice were analyzed at 4, 7 and 20 days p.i. with 1 × 10^6^ iRBCs. Naïve mice were used as controls (day 0). The data were expressed as means ± SD (*n* = 3–5) of one representative experiment out of three. Significant differences were observed for the (*) indicated groups with *p* < 0.05, using the Mann Whitney U test (NS, not significant).
(A) Contour plots show T-bet and Bcl6 expression in CD4^+^ cells. T-bet^+^Bcl6^+^ cell percentages in CD4^+^ cells are shown in the column bar graphs. Histograms show T-bet and Bcl6 expression in relation to FMO and isotype controls.
(B) Histograms show T-bet and Bcl6 expression in relation to FMO and isotype controls.
(C) Contour plots show PD1 and CXCR5 expression in CD4^+^ cells.
(D) Foxp3^+^CD4^+^ cell numbers per spleen were determined by flow cytometry.
(E) Contour plots show CD25 and CD122 expression in Foxp3^+^CD4^+^ cells. CD25^+^CD122^+^Foxp3^+^ cell percentages in CD4^+^ cells and CD25^+^CD122^+^Foxp3^+^CD4^+^ cell numbers per spleen are shown in the column bar graph.
(F) Histograms show P2X7 and CD39 expression in CD4^+^, T-bet^+^Bcl6^+^CD4^+^ and T-bet^-^Bcl6^-^CD4^+^ cells. The MFIs of P2X7 and CD39 expression are shown in the column bar graphs.
(TIF)

S4 Fig CD4 TE, TEM and TCM cell numbers per spleen and phenotypic characterization of IFNγ- and IL-10-producing cells in chronically infected B6 and *P2rx7*^-/-^ mice. (A-B) B6 and *P2rx7*^-/-^ female mice were analyzed at 20 and 30 days p.i. with 1 × 10^6^ *Pc*-iRBCs. Naïve mice were used as controls (day 0). The data were expressed as means ± SD (*n* = 3) of one representative experiment out of three. Significant differences were observed for the (*) B6 and *P2rx7*^-/-^ groups with *p* < 0.05, using the Mann Whitney U test (NS, not significant).
(A) The gating strategy used to define CD4^+^ cell subsets is shown. CD4 T_E_ (CD44^hi^IL-7Rα^-^), T_EM_ (CD44^hi^IL-7Rα^+^CD62L^lo^) and T_CM_ (CD44^hi^IL-7Rα^+^CD62L^hi^) cell numbers per spleen were determined by flow cytometry.
(B) Contour plots (left) show IFNγ and IL-10 expression in CD4^+^ cells. The gate strategy to identify T_E_, T_EM_ and T_CM_ cells is shown in the contour plot and histogram (upper right), according to CD44, CD127 and CD62L expression. IFNγ^+^IL-10^-^CD4^+^ and IFNγ^-^IL-10^+^CD4^+^ cells were analyzed using the same markers (middle and lower right).
(TIF)

S5 Fig Characterization of splenic CD4 TE/EM and TCM cells from chronically infected B6 and *P2rx7*^-/-^ mice. (A-C) B6 and *P2rx7*^-/-^ female mice were analyzed at 20 and 30 days p.i. with 1 × 10^6^ *Pc*-iRBCs. Naïve mice were used as controls (day 0). The data were expressed as means ± SD (*n* = 3–4) of one representative experiment out of three. Significant differences were observed for the (*) indicated groups with *p* < 0.05, using the Mann Whitney U test (NS, not significant).
(A) Contour plots show naïve (CD44^-^CD62L^hi^), CD4 T_E/EM_ (CD44^+^CD62L^lo^) and T_CM_ (CD44^+^CD62L^hi^) cells. Percentages of each CD4^+^ cell subset are shown. Histograms show T-bet expression in CD4^+^ cell subsets. FMO controls are shown in the Fig 5E. The MFIs of T-bet expression are shown in the scatter plots.
(B) Histograms show P2X7 expression in naïve (CD44^-^CD62L^hi^), CD4 T_E/EM_ (CD44^+^CD62L^lo^) and T_CM_ (CD44^+^CD62L^hi^) cells. The MFIs of P2X7 expression are shown in the column bar graph.
(C) Contour plots show CD44 and CD62L expression in PD1^hi^Bcl6^+^CD4^+^ and PD1^-^Bcl6^-^CD4^+^ cells.
(TIF)

S6 Fig Splenic CD4 T cell populations in *Cd4*^-/-^ mice adoptively transferred with B6 or *P2rx7*^-/-^ CD4 cells and infected with Pc parasites. (A-B) Naïve CD4^+^ cells from B6 and *P2rx7*^-/-^ mice were transferred into *Cd4*^-/-^ mice that were infected with 1 × 10^6^ iRBCs 7 days later. Splenic CD4^+^ cells were analyzed at 7 and 30 days p.i. Naïve mice were used as controls (day 0). The data were expressed as means ± SD (*n* = 3–5) of one representative experiment out of three. Significant differences were observed for the (*) B6 and *P2rx7*^-/-^ groups with *p* < 0.05, using the Mann Whitney U test (NS, not significant).
(A) CD4^+^ cell numbers per spleen were determined by flow cytometry.
(B) CD4 T_E_ (CD44^+^IL-7Rα^-^), T_EM_ (CD44^+^IL-7Rα^+^CD62L^lo^) and T_CM_ (CD44^+^IL-7Rα^+^CD62L^hi^) cell numbers per spleen were determined by flow cytometry.
(TIF)
