# Supplementary figures and images for: P2X7 receptor drives Th1 cell differentiation and controls the follicular helper T cell population to protect against Plasmodium chabaudi malaria
Source: PLoS Pathog. 2017 Aug 31;13(8):e1006595. doi: 10.1371/journal.ppat.1006595 (PMC5597262; doi:10.1371/journal.ppat.1006595)

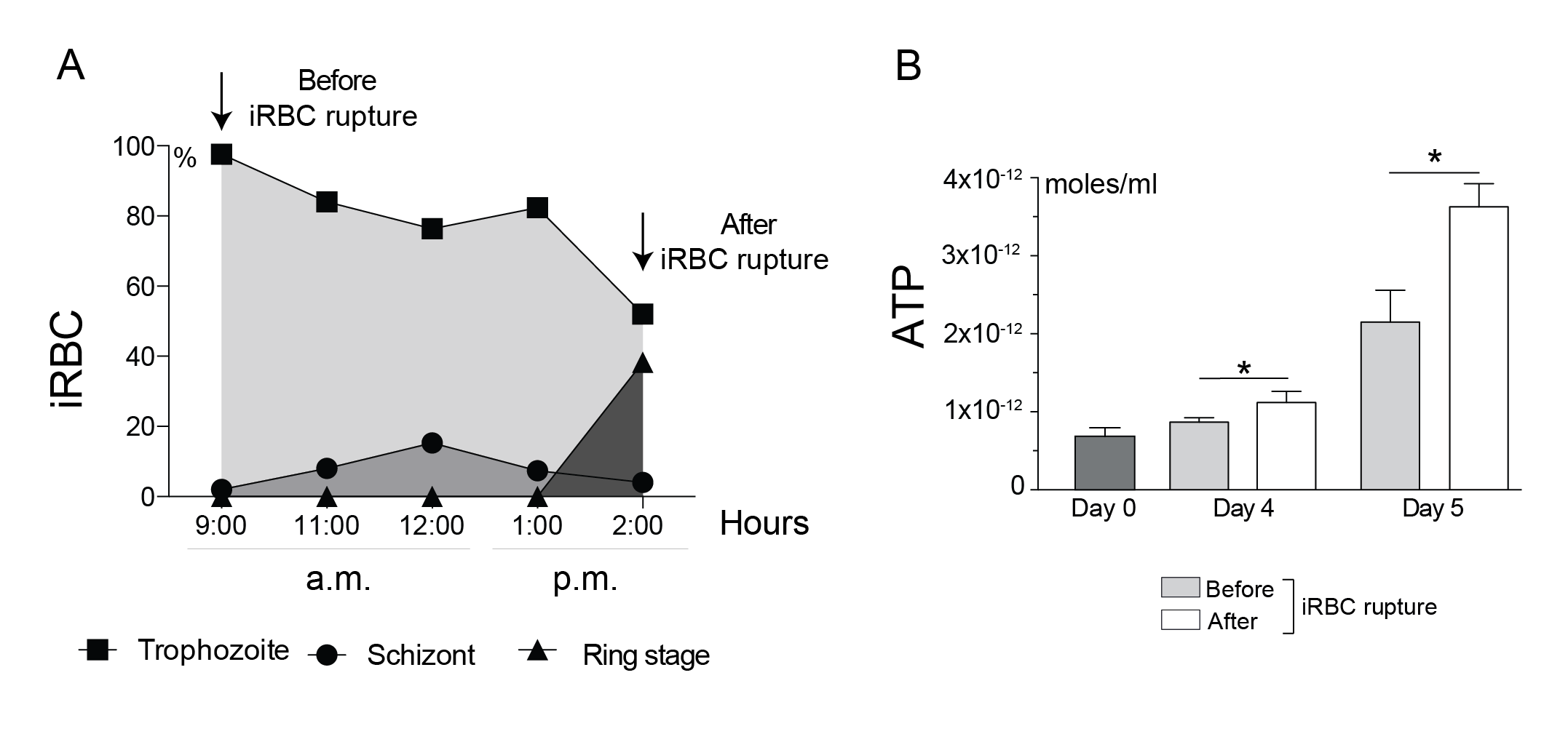

Supplement: S1 Fig — (A) The percentages of trophozoites, shizonts and ring stages were determined at 5 days p.i. with 1 × 10^6^ iRBCs (n = 3). Arrows indicate the time at which blood samples were collected. (B) B6 and P2rx7-/- female mice were analyzed at 4 and 5 days p.i. with 1 × 10^6^ iRBCs. Naïve mice were used as controls (day 0). The data were expressed as means ± SD (n = 3) of one representative experiment out of three. Significant differences were observed for the (*) indicated groups with p < 0.05, using the Mann Whitney U test. ATP concentrations were determined by bioluminescence in B6 mouse serum before and after iRBC rupture. The blood samples were collected at 9 a.m. (6.5 ± 0.5% iRBC at day 4 p.i. and 10.5 ± 1.5% iRBCs at 5 days p.i.; >95% trophozoites and schizonts) and 2 p.m. (12.0 ± 1.2% iRBC at day 4 p.i. and 25.0 ± 3.7% iRBCs at 5 days p.i.; >95% ring forms). (TIF) [file ppat.1006595.s001.tif]

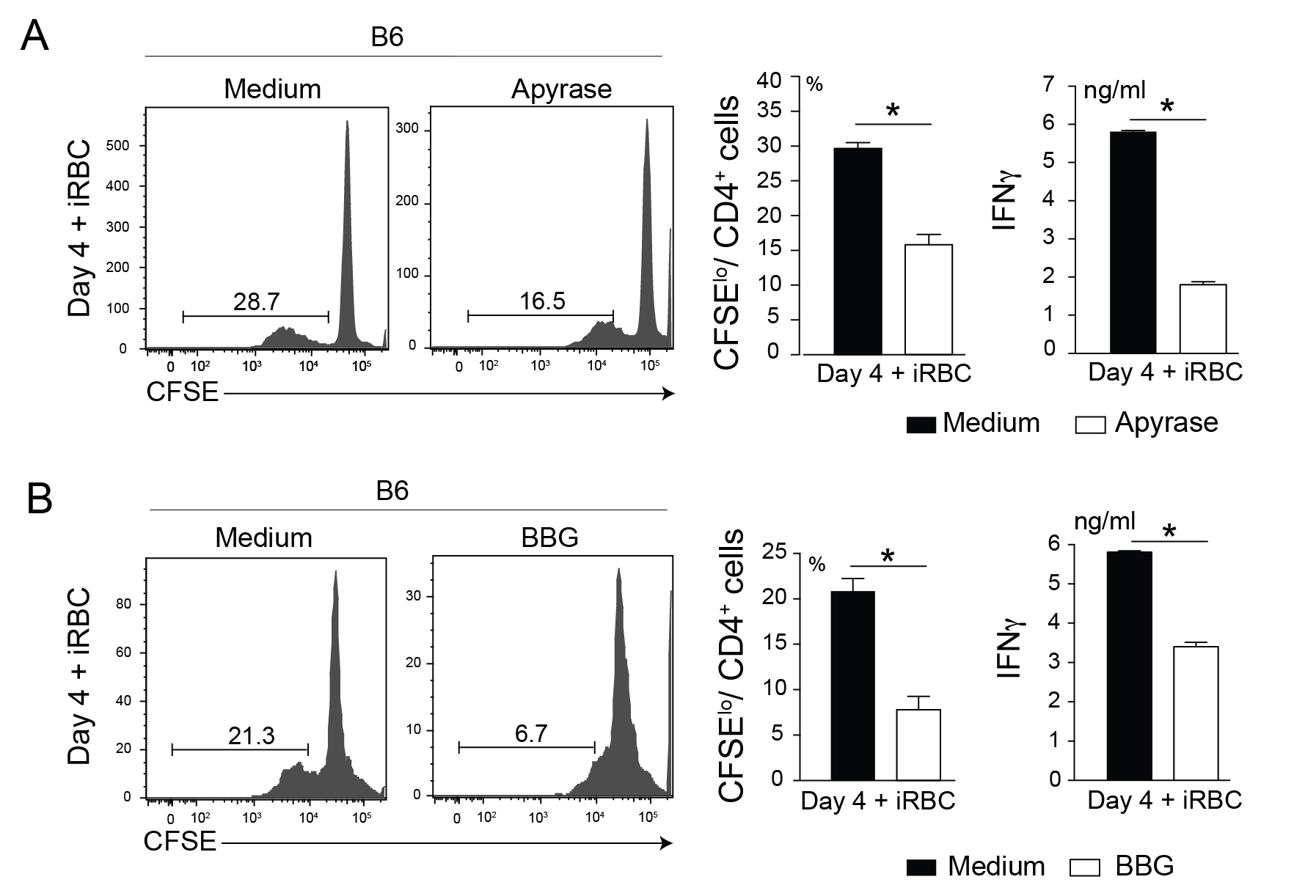

Supplement: S2 Fig — (A-B) B6 mice were analyzed at 4 days p.i. with 1 × 10^6^ Pc-iRBCs. The data were expressed as means ± SD (n = 3) of one representative experiment out of three. Significant differences were observed for the (*) indicated groups with p < 0.05, using the Mann Whitney U test (NS, not significant). (A) CFSE-stained splenocytes were stimulated with iRBCs (1 splenocyte/ 4 iRBCs) in the presence or not of apyrase. CFSEloCD4+ cell percentages are shown in the column bar graph. IFNγ concentrations were determined by ELISA in the culture supernatants. (B) CFSE-stained splenocytes were stimulated with iRBCs (1 splenocyte/ 4 iRBCs) in the presence or not of BBG. CFSEloCD4+ cell percentages are shown in the column bar graph. IFNγ concentrations were determined by ELISA in the culture supernatants. (TIF) [file ppat.1006595.s002.tif]

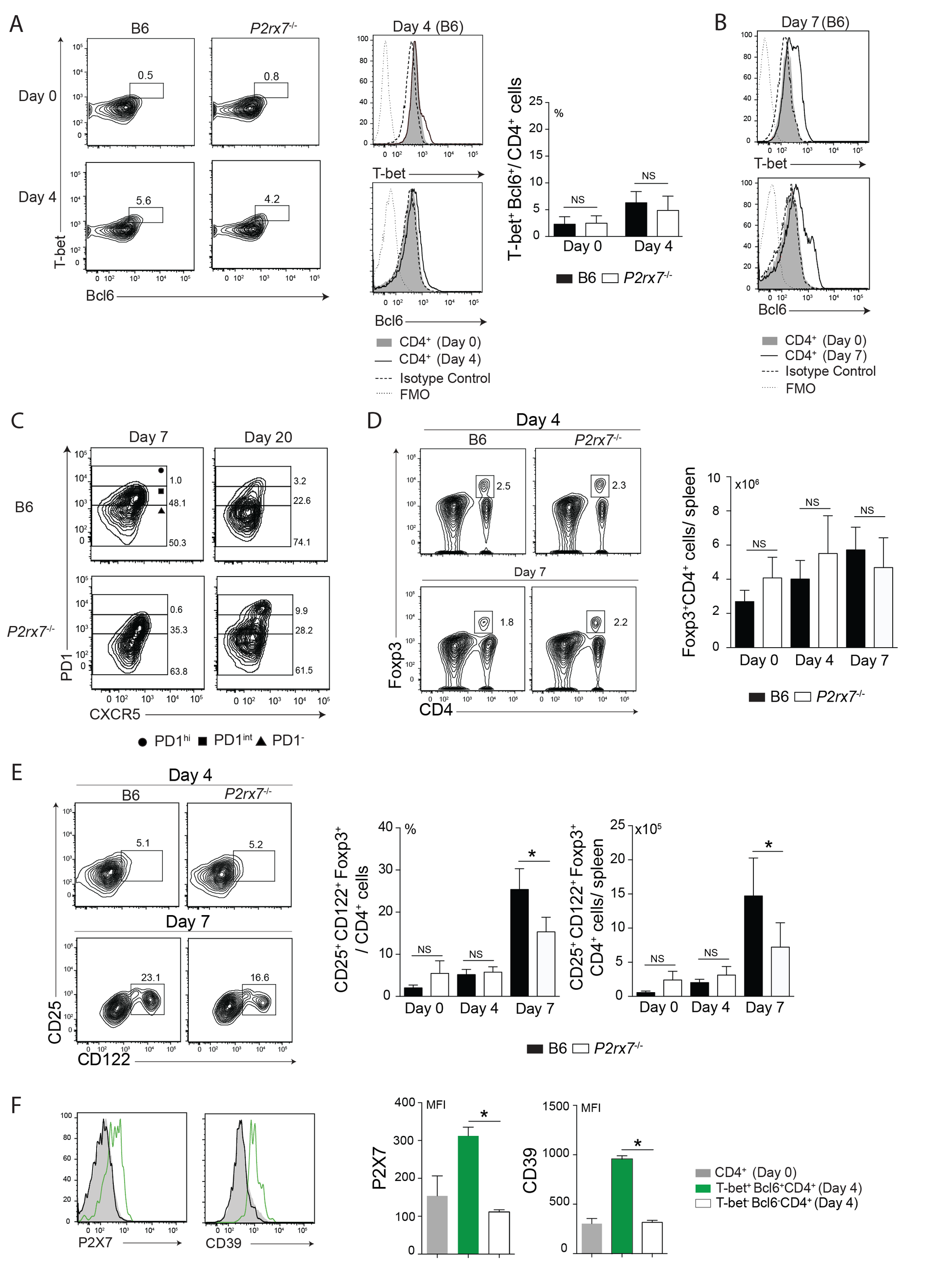

Supplement: S3 Fig — (A-F) B6 and P2rx7-/- female mice were analyzed at 4, 7 and 20 days p.i. with 1 × 10^6^ iRBCs. Naïve mice were used as controls (day 0). The data were expressed as means ± SD (n = 3–5) of one representative experiment out of three. Significant differences were observed for the (*) indicated groups with p < 0.05, using the Mann Whitney U test (NS, not significant). (A) Contour plots show T-bet and Bcl6 expression in CD4+ cells. T-bet+Bcl6+ cell percentages in CD4+ cells are shown in the column bar graphs. Histograms show T-bet and Bcl6 expression in relation to FMO and isotype controls. (B) Histograms show T-bet and Bcl6 expression in relation to FMO and isotype controls. (C) Contour plots show PD1 and CXCR5 expression in CD4+ cells. (D) Foxp3+CD4+ cell numbers per spleen were determined by flow cytometry. (E) Contour plots show CD25 and CD122 expression in Foxp3+CD4+ cells. CD25+CD122+Foxp3+ cell percentages in CD4+ cells and CD25+CD122+Foxp3+CD4+ cell numbers per spleen are shown in the column bar graph. (F) Histograms show P2X7 and CD39 expression in CD4+, T-bet+Bcl6+CD4+ and T-bet-Bcl6-CD4+ cells. The MFIs of P2X7 and CD39 expression are shown in the column bar graphs. (TIF) [file ppat.1006595.s003.tif]

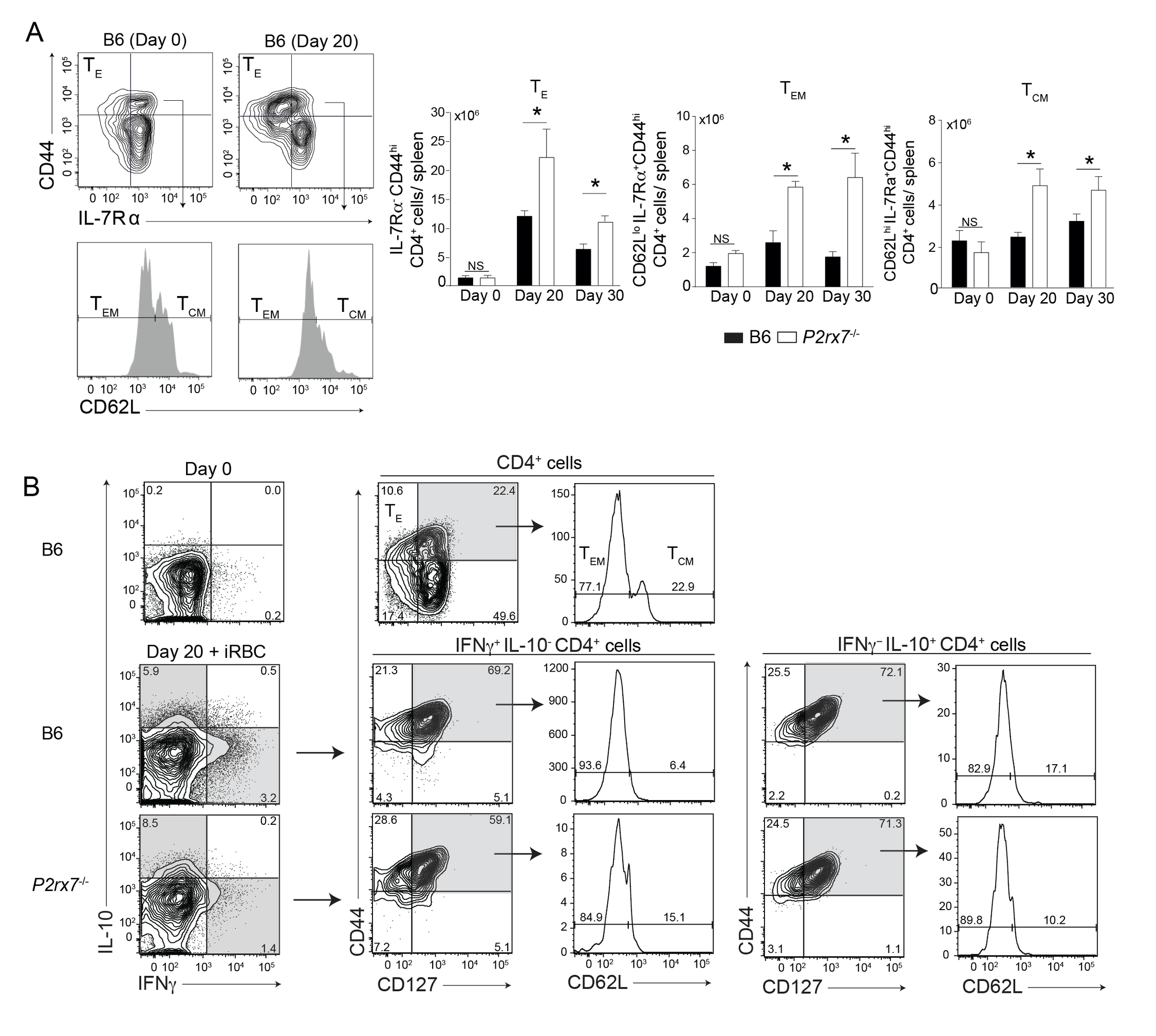

Supplement: S4 Fig — (A-B) B6 and P2rx7-/- female mice were analyzed at 20 and 30 days p.i. with 1 × 10^6^ Pc-iRBCs. Naïve mice were used as controls (day 0). The data were expressed as means ± SD (n = 3) of one representative experiment out of three. Significant differences were observed for the (*) B6 and P2rx7-/- groups with p < 0.05, using the Mann Whitney U test (NS, not significant). (A) The gating strategy used to define CD4+ cell subsets is shown. CD4 TE (CD44hiIL-7Rα-), TEM (CD44hiIL-7Rα+CD62Llo) and TCM (CD44hiIL-7Rα+CD62Lhi) cell numbers per spleen were determined by flow cytometry. (B) Contour plots (left) show IFNγ and IL-10 expression in CD4+ cells. The gate strategy to identify TE, TEM and TCM cells is shown in the contour plot and histogram (upper right), according to CD44, CD127 and CD62L expression. IFNγ+IL-10-CD4+ and IFNγ-IL-10+CD4+ cells were analyzed using the same markers (middle and lower right). (TIF) [file ppat.1006595.s004.tif]

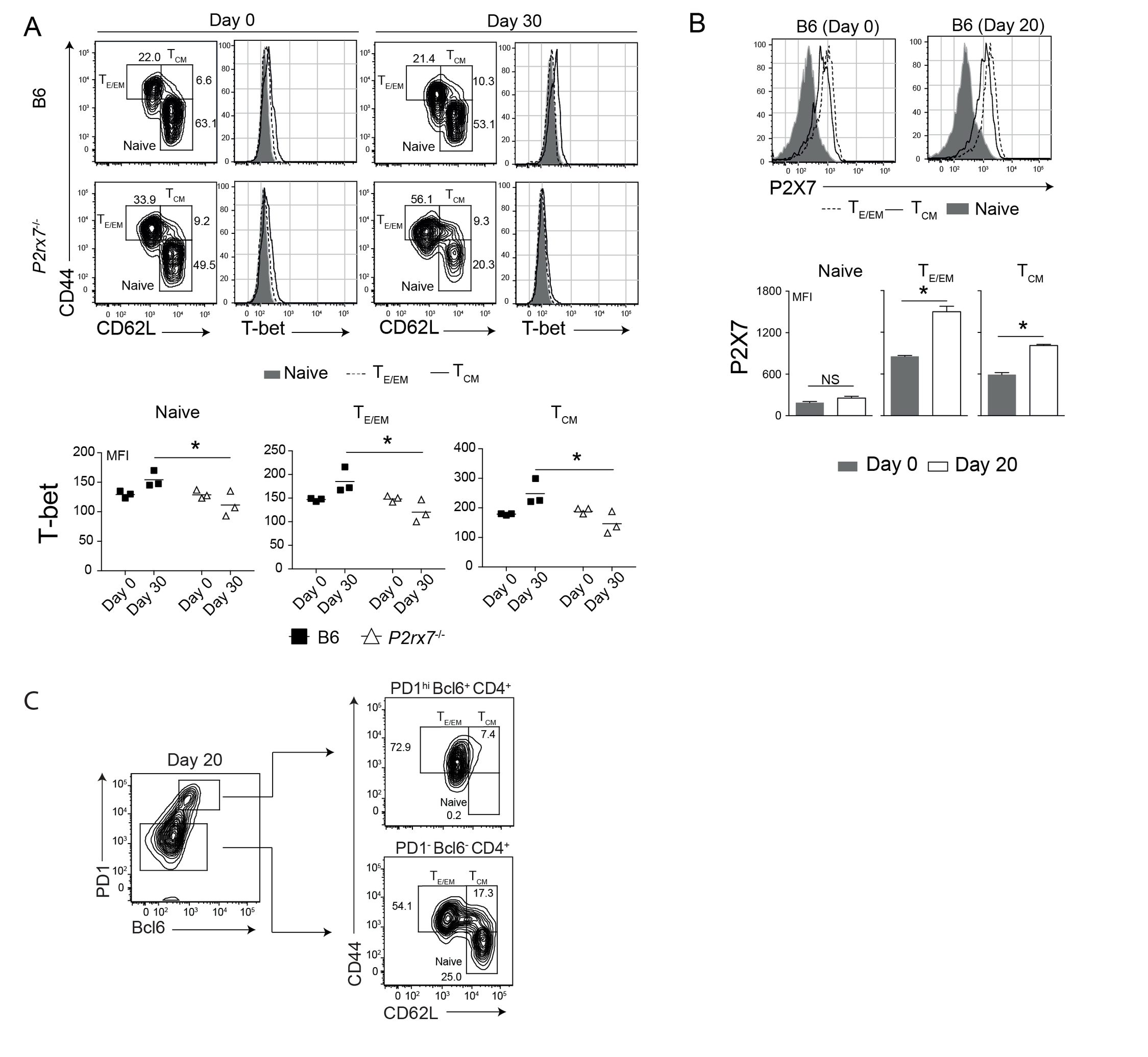

Supplement: S5 Fig — (A-C) B6 and P2rx7-/- female mice were analyzed at 20 and 30 days p.i. with 1 × 10^6^ Pc-iRBCs. Naïve mice were used as controls (day 0). The data were expressed as means ± SD (n = 3–4) of one representative experiment out of three. Significant differences were observed for the (*) indicated groups with p < 0.05, using the Mann Whitney U test (NS, not significant). (A) Contour plots show naïve (CD44-CD62Lhi), CD4 TE/EM (CD44+CD62Llo) and TCM (CD44+CD62Lhi) cells. Percentages of each CD4+ cell subset are shown. Histograms show T-bet expression in CD4+ cell subsets. FMO controls are shown in the Fig 5E. The MFIs of T-bet expression are shown in the scatter plots. (B) Histograms show P2X7 expression in naïve (CD44-CD62Lhi), CD4 TE/EM (CD44+CD62Llo) and TCM (CD44+CD62Lhi) cells. The MFIs of P2X7 expression are shown in the column bar graph. (C) Contour plots show CD44 and CD62L expression in PD1hiBcl6+CD4+ and PD1-Bcl6-CD4+ cells. (TIF) [file ppat.1006595.s005.tif]

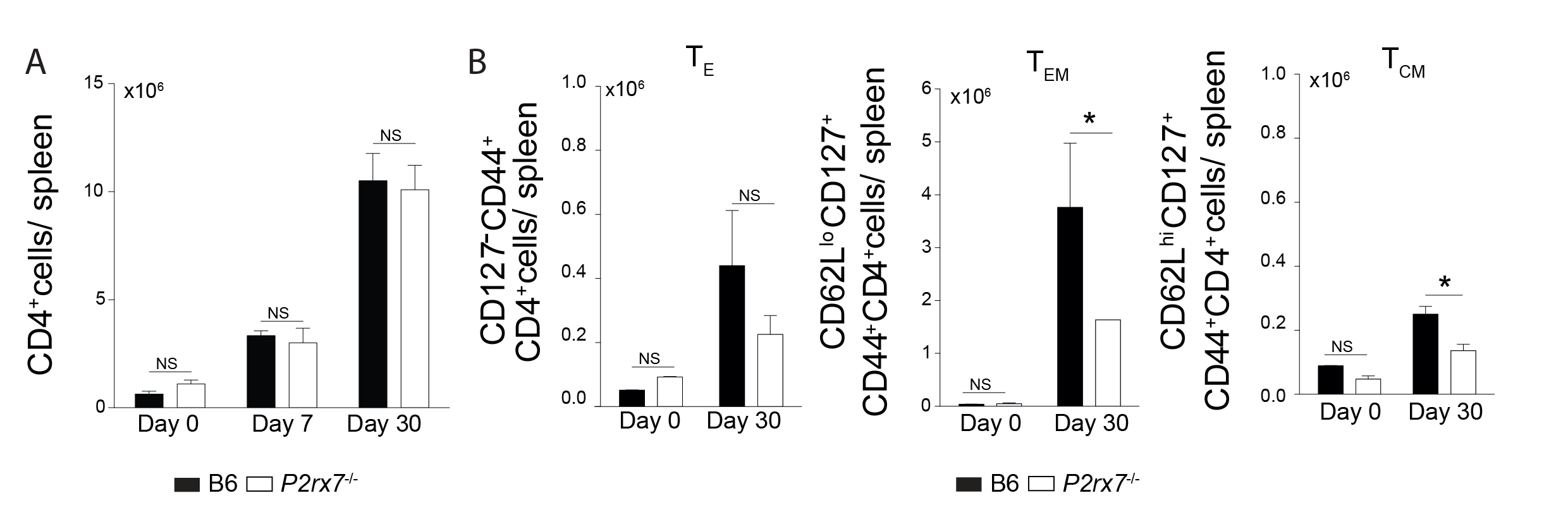

Supplement: S6 Fig — (A-B) Naïve CD4+ cells from B6 and P2rx7-/- mice were transferred into Cd4-/- mice that were infected with 1 × 10^6^ iRBCs 7 days later. Splenic CD4+ cells were analyzed at 7 and 30 days p.i. Naïve mice were used as controls (day 0). The data were expressed as means ± SD (n = 3–5) of one representative experiment out of three. Significant differences were observed for the (*) B6 and P2rx7-/- groups with p < 0.05, using the Mann Whitney U test (NS, not significant). (A) CD4+ cell numbers per spleen were determined by flow cytometry. (B) CD4 TE (CD44+IL-7Rα-), TEM (CD44+IL-7Rα+CD62Llo) and TCM (CD44+IL-7Rα+CD62Lhi) cell numbers per spleen were determined by flow cytometry. (TIF) [file ppat.1006595.s006.tif]
